# Quality of home visits by community health workers in primary care and associated factors *

**DOI:** 10.1590/1518-8345.7203.4398

**Published:** 2024-11-25

**Authors:** Marciane Kessler, Elaine Thumé, Luiz Augusto Facchini, Luiza Carolina Moro, Elaine Tomasi

**Affiliations:** ^1^ Universidade Regional Integrada do Alto Uruguai e das Missões, Curso de Enfermagem, Erechim, RS, Brazil.; ^2^ Scholarship holder at the Conselho Nacional de Desenvolvimento Científico e Tecnológico (CNPq), Brazil.; ^3^ Universidade Federal de Pelotas, Pelotas, RS, Brazil.; ^4^ Universidade Federal de Pelotas, Departamento de Medicina Social, Pelotas, RS, Brazil.; ^5^ Associação Hospitalar Lenoir Vargas Ferreira, Chapecó, SC, Brazil.

**Keywords:** Family Health Strategy, Community Health Workers, House Calls, Quality Indicators, Health Care, Health Equity, Community Health Nursing

## Abstract

to evaluate community health workers’ and quality of home visits associated factors.

a cross-sectional study of 38,865 teams and 140,444 Primary Care Access and Quality Improvement Program users. We established the “quality of home visits” and its association with the characteristics of the municipalities, teams and individuals, estimated by the prevalence ratio and 95% confidence intervals.

139,362 (99.2%) users said the health team had community workers. The prevalence of quality was 51.9%. After adjustments, the prevalence was higher in the Northeast (Prevalence Ratio: 1.19 [1.18-1.21]), smaller municipalities (PR: 1.06 [1.03-1.09]), among teams with area definition (PR: 1.15 [1.06-1.25]), evaluation of indicators (PR: 1.14 [1.12-1.17]) and user satisfaction (PR: 1.11 [1.08-1.13]), that considered risk and vulnerability criteria for defining the number of people under responsibility (PR: 1.05 [1.04-1.07]) and without an uncovered population of workers (PR: 1.03 [1.01-1.04]); among users with chronic diseases (PR: 1.02 [1.01-1.05]) and with someone at home with walking difficulties (PR: 1.05 [1.02-1.07]).

the importance of organizing the teams’ work process for the quality of the worker’s home visits and their role in health equity is highlighted.

## Highlights

**(1)** Half of the users receive a home visit from the health worker with adequate quality.

**(2)** Quality is higher in the Northeast, smaller municipalities and teams with a defined area.

**(3)** Quality has increased with the evaluation of indicators and user satisfaction.

**(4)** Considering risks and vulnerability when defining territory increased quality.

**(5)** Quality was higher among users with chronic diseases and disabilities.

## Introduction

 Over the last two decades, there has been growing interest in and efforts to evaluate the Family Health Strategy (FHS) progress in Brazil ^(^ 1 ^-^ 5 ^)^ . The aim is to demonstrate its effectiveness in achieving universality, comprehensiveness, longitudinality, and equity in health ^(^ 3 ^)^ and positively impacting the population’s health. 

 Family health teams increased from 2,054 in 1998 ^(^ 6 ^)^ to 43,508 in 2019 ^(^ 7 ^)^ . In the same period, the population covered by the FHS went from 4.4% (seven million Brazilians) ^(^ 6 ^)^ to 64.2% (134 million) ^(^ 7 ^)^ , with coverage being higher in Brazil’s poorest regions and areas. The expansion of the FHS in the country has helped the Unified Health System (SUS) to become one of the largest public health systems in the world, with universal access to health and based on primary care ^(^ 8 ^)^ . 

 Alongside the growth in FHS coverage in Brazil, the literature has shown the model’s impact on reducing infant mortality ^(^ 9 ^-^ 10 ^)^ , hospitalizations for primary care-sensitive conditions ^(^ 10 ^)^ , the control of cardiovascular diseases ^(^ 11 ^)^ , and social inequality in mortality among the older adult ^(^ 2 ^)^ . The FHS has also contributed to increased coverage of programmatic actions, such as prenatal care ^(^ 12 ^)^ ; cervical cancer prevention ^(^ 13 ^)^ , and greater access to and use of health services, especially among socially vulnerable people ^(^ 1 ^-^ 3 ^)^ . In addition, various studies have shown the importance of the FHS in increasing the quality of the services provided by primary care ^(^ 3 ^-^ 4 ^)^ . However, no national studies have evaluated the quality of services offered by Community Health Workers (CHWs) during home visits. 

 It should be noted that the positive results of the FHS were only possible due to its organizational characteristics and its replacement of the traditional model of basic care. The FHS has a care model aimed at universal, comprehensive, continuous, and equitable care ^(^ 1 ^,^ 3 ^)^ , with a focus on health promotion, disease prevention, early diagnosis, and treatment; and includes the community health worker as a member of the team, who works in a delimited geographical area, registering and monitoring the population in the territory. 

 The number of workers per team should be defined according to the characteristics of the territory’s population, considering criteria of demography, epidemiology, economics and social issues; and in dispersed territorial areas, with the presence of risk and social vulnerability, 100% population coverage is recommended ^(^ 14 ^)^ . In 2020, 257,770 community workers were part of the FHS, with a population coverage of 61.1% in the country ^(^ 7 ^)^ . 

 The CHW, who maintains a strong daily link with the nursing team and is managed by the nurse ^(^ 15 ^-^ 16 ^)^ , is also one of the people responsible for the results achieved by the FHS - when they carry out quality work - being the link between health services and the community and promoting bonds between users and professionals ^(^ 17 ^-^ 18 ^)^ . One of the worker’s main activities is the home visit ^(^ 18 ^)^ , through which they register the family, help the team identify areas and situations of risk, provide guidance on health promotion and protection, mobilize the community to seek favorable health conditions and participate in groups, notify the services of diseases that need monitoring, refer people with health needs to the centers, monitor the treatment and rehabilitation of sick people at home ^(^ 17 ^-^ 19 ^)^ . 

 The characterization of quality in this study was built from the perspective of integrality or completeness of activities through an approach that considers the whole, the psychological, biological, and sociocultural aspects, together with the offer of humanized actions aimed at health promotion, protection, prevention, recovery, and rehabilitation. One of the most important principles of the SUS is comprehensiveness, and it is also an essential attribute in assessing the quality of primary health care (PHC), a preferred space for its expression ^(^ 4 ^)^ . The quality of PHC is the result of well-organized and managed services, supported by sufficient funding to provide adequate supplies, human resources, infrastructure and medicines ^(^ 1 ^,^ 4 ^)^ . 

Given this context, this study aimed to assess community health workers’ quality of home visits and associated factors.

## Method

### Study design

This is a cross-sectional study based on health services that was part of the External Evaluation phase of primary care teams throughout the country, using data from the third cycle of the Program for Improving Access and Quality (PMAQ, its acronym in Portuguese), established by the Ministry of Health to subsidize funding for performance in primary care from 2011 to 2018.

The PMAQ was launched in 2011 to encourage managers and teams to improve the quality of health services offered to citizens in the territory, through federal financial incentives for participating municipalities that improved quality standards. To this end, a set of strategies was proposed for qualifying, monitoring, and evaluating the work of health teams.

The study was coordinated by more than 50 Brazilian higher education institutions and led by: the Oswaldo Cruz Foundation (FIOCRUZ), Federal University of Minas Gerais (UFMG), Federal University of Rio Grande do Norte (UFRN), Federal University of Pelotas (UFPel), Federal University of Bahia (UFBA), Federal University of Mato Grosso do Sul (UFMS), Federal University of Pará (UFPA), Federal University of Piauí (UFPI) and Federal University of Sergipe (UFS).

### Selection criteria

Municipalities, health teams, and team users from all over the country who voluntarily joined the PMAQ 2017/18 were included. Users were excluded if they were under 18 years of age, attending the health center for the first time, or it had been more than 12 months since they last attended.

### Population and sample

Cycle 3 of the PMAQ evaluation included 5,324 municipalities, 28,939 primary care centers, 38,865 teams, and 140,444 users. One physician, nurse, or dentist from each team took part in the study, and the respondent did not have to be the team coordinator. Among users, sampling was by convenience, i.e., users attending the health service on the day of the interviewer’s visit.

### Study variables

 The outcome “quality of the home visit provided by the community health worker” was constructed considering the completeness of the activities or their full implementation by the CHWs, as mentioned previously ^(^ 4 ^)^ . In this study, an indicator was structured based on users’ positive responses to the following questions: “During the visit, does the health worker ask about the family’s health problems?, Advises on care and disease prevention?, Inform about the actions of the primary care center/health center near your home?, Deliver documents such as appointments and exams?, and Carry out actions to combat the *Aedes aegypti* mosquito?”. All the questions were answered dichotomously (yes/no). 

The exposure variables used to verify the association with the adequate quality of the worker’s visit were: a) municipal variables (secondary source) - geopolitical region (North, Northeast, Midwest, Southeast and South), population size, in inhabitants (up to 10,000; 10,001-30,000; 30,001-100,000; 100,001-300,000; more than 300,000) and population coverage (up to 50%; 50.1%-75%; 75.1%-99.000; 100%). 000) and population coverage of the FHS (up to 50%; 50.1%-75%; 75.1%-99.9%; 100%); b) team variables (interview with professional) - definition of the team’s catchment area (yes; no); whether the management considered risk and vulnerability criteria when defining the number of people under the team’s responsibility (yes; no); whether the team monitors and analyzes health indicators and information (yes; no); whether the team evaluates user satisfaction (yes; no); and whether there is an undiscovered population of workers in the territory (yes; no); and c) variables of the individual (interview with the service user) - sex (male; female), age in complete years (18-39; 40-59; 60 or over), self-reported ethnicity/skin color (indigenous/brown/black/other and white); chronic health conditions reported by medical diagnosis (none; hypertension or diabetes; hypertension and diabetes), family member with walking difficulties investigated by the question: “Do you have anyone at home with walking difficulties who needs home care?” (yes; no), the presence of pregnancy in the last two years investigated by the question: “Have you been pregnant in the last two years?” (yes; no) and the presence of children up to two years old, through the question: “Do you have a child up to two years old?” (yes; no).

### Instrument used to collect information

 This study used information from Modules I, II, and III of the PMAQ Cycle 3 collection instrument ^(^ 20 ^)^ . Module I refers to observation at the health center, with questions about infrastructure; Module II refers to an interview with a professional about the work process of the primary care team and checking documents at the health center; and Module III refers to an interview with users at the health center evaluated. 

### Data collection

The questionnaire was administered in the health centers on a date agreed upon by municipal management. Module I contained data from the interviewer’s observation, Module II was answered by a physician, nurse, or dentist, and Module III as completed by users in the center on the day of the external evaluation.

From 2017 to 2018, data was collected by around 1,000 trained interviewers and supervisors in all states, using an electronic instrument on tablets, which was then automated and transmitted to the Ministry of Health. Data quality control was carried out by supervising data collection and using an electronic validator to check the consistency of responses.

### Data analysis

 In addition to the descriptive analyses, the prevalence of the outcome was calculated according to the characteristics of the municipalities, the team and the individual. The associated factors were analyzed using Poisson regression with robust variance adjustment to estimate the prevalence ratios (PR) with their respective 95% confidence intervals (95%CI). The adjusted analysis used a hierarchical model based on the social determination of health, valuing the characteristics of the context as macro-determinants of the variables located at intermediate and proximal levels to the outcome ^(^ 1 ^-^ 2 ^)^ . The first level included the region variable; the second level included variables related to the municipality; the third level included variables related to the team; the fourth level included those related to the individual’s demographic and social characteristics; and finally, the fifth level included individual health conditions. Backward selection was applied by hierarchical levels, eliminating all variables with a p-value ≥0.20 from the model. The criterion used to define the reference category (1.00) for each variable was the lowest value observed to highlight the probability of an increase in the occurrence of the outcome. Statistical significance was verified using the Wald and heterogeneity tests, considering a 5% level. The statistical package Stata 14.0 (StataCorp LP, College Station, United States) was used to analyze the data. 

### Ethical aspects

The project was submitted to and approved by the Federal University of Pelotas research ethics committee in 2017 under protocol number 80341517.8.1001.5317, with opinion number 2.453.320. All participants signed an informed consent form. The authors declare no conflicts of interest regarding the study’s subject.

## Results

**Figure 1 -:**
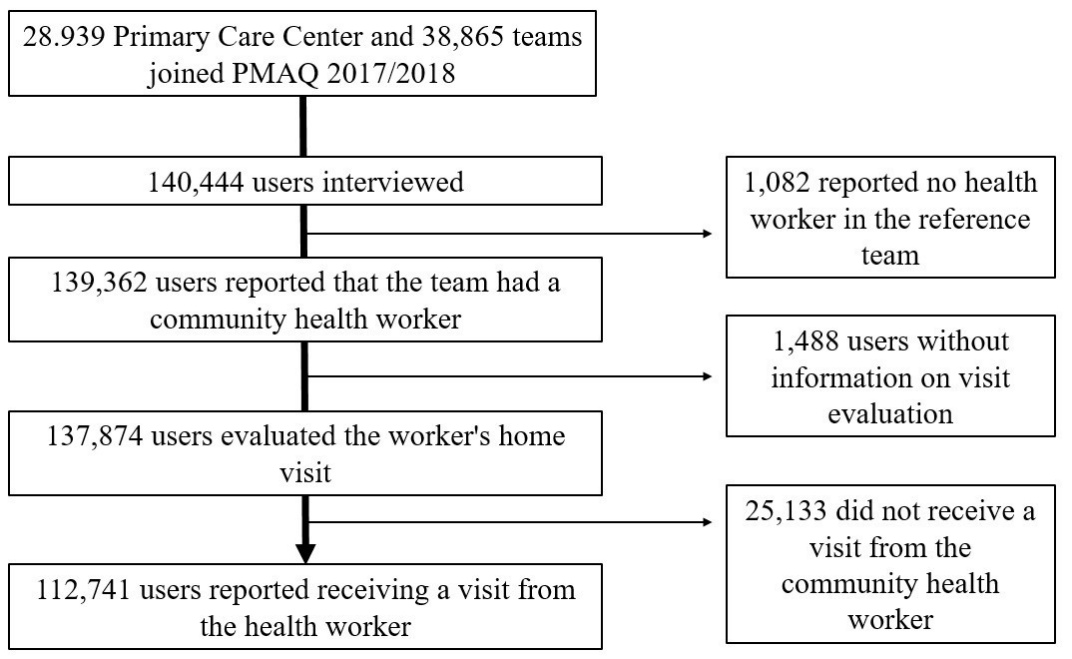
Selection process of the sample included in the analysis. PMAQ* Cycle 3. Brazil, 2017-2018

 Of all the users interviewed, 139,362 (99.2%) said their health team had community workers, as shown in Figure 1 . 

*PMAQ = Program for Improving Access and Quality

 Among these, 37.3% of users were concentrated in the Northeast and 33.4% in the Southeast; 40.2% lived in municipalities with up to 30,000 inhabitants; 45.1% in municipalities with 100% coverage; 99.2% of users belonged to teams where there was a definition of the team’s catchment area, 80.0% belonged to teams that considered risk and vulnerability criteria when defining the number of people under their responsibility, 88.4% belonged to teams that monitored and analyzed health indicators and information, 87.0% belonged to teams that evaluated user satisfaction and 39.8% of users were interviewed in services whose teams had areas in the territory discovered by CHWs ( Table 1 ). 

**Table 1 -:** Distribution of the sample of users according to the characteristics of the municipalities and teams (n = 139,362). PMAQ* Cycle 3. Brazil, 2017-2018

**Variable**	**Total sample**	
	**n**	**%**
**Total**	139,362	100.0
**Characteristics of the municipalities**		
Region		
South	19,971	14.3
Southeast	46,609	33.4
Midwest	11,723	8.4
Northeast	51,956	37.3
North	9,103	6.5
Size of municipality (inhabitants)		
Up to 10,000	18,198	13.1
10,001-30,000	37,795	27.1
30,001-100,000	33,102	23.8
100,001-300,000	18,783	13.5
More than 300,000	31,484	22.6
FHS ^†^ coverage (%)		
Up to 50	20,535	14.7
50,1-75,0	26,734	19.2
75,1-99,9	29,256	21.0
100,0	62,837	45.1
**Team characteristics**		
Definition of the team’s catchment area		
Yes	138,222	99.2
No	1,140	0.8
Consideration of risk and vulnerability criteria to define the number of people under the team’s responsibility ^‡^		
Yes	101,270	80.0
No	25,293	20.0
Monitoring and analysis of health indicators and information		
Yes	123,154	88.4
No	16,208	11.6
User satisfaction assessment		
Yes	121,221	87.0
No	18,141	13.0
Population uncovered by health workers		
Yes	55,478	39.8
No	83,884	60.2

*PMAQ Program for Improving Access and Quality

^†^ FHS Family Health Strategy

^‡^ 12,799 missing/no response

 Among the users’ characteristics, 78.4% were female, 77.9% were between 18 and 59 years old, and 68.1% self-reported ethnicity/skin color as indigenous, brown, black, and others. Among the users, 28.3% reported being hypertensive or diabetic, and 9.1% had both conditions; 7.8% reported having someone with walking difficulties in the house where they live, 19.2% of the users reported pregnancy in the last two years, and 11.2% of the users reported having a child up to 2 years old ( Table 2 ). 

**Table 2:** - Distribution of the user sample according to individual user characteristics (n = 139,362). PMAQ* Cycle 3. Brazil, 2017-2018

**Variable**	**Total sample**	
	**n**	**%**
Total	139,362	100.0
Individual characteristics		
Sex		
Female	109,294	78.4
Male	30,068	21.6
Age (years)		
18-39	60,697	43.6
40-59	47,758	34.3
60 or more	30,907	22.2
Ethnicity/Skin color ^†^		
White	43,937	31.9
Indigenous/Brown/Black/Other	93,645	68.1
Chronic Condition ^‡^		
None	86,779	62.6
Hypertension or diabetes	39,276	28.3
Hypertension and diabetes	12,613	9.1
Someone with walking difficulties in the house ^§^		
Yes	10,878	7.8
No	128,342	92.2
Pregnancy in the last 2 years ^ǁ^		
Yes	20,953	19.2
No	88,358	80.8
Child up to 2 years old ^⁋^		
Yes	15,621	11.2
No	123,521	88.8

*PMAQ Program for Improving Access and Quality

^†^ 1,780 missing/no response

^‡^ 694 missing/no response

^§^ 142 missing/no response

^ǁ^ 30,051 missing/no response

^⁋^ 220 missing/no response

**Figure 2 -:**
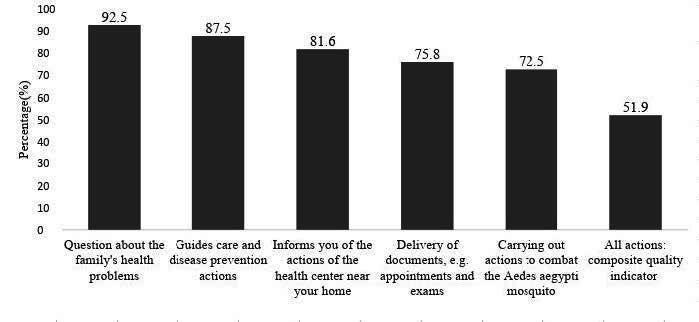
Proportion of actions carried out during home visits by community workers and composite quality indicator (n = 112,741). PMAQ* Cycle 3. Brazil, 2017-2018

^*^ PMAQ = Program for Improving Access and Quality

 A total of 137,874 users assessed whether they received a visit from the health worker among teams with this professional, and 112,741 (81.2%) said they did ( Figure 1 ). Among the actions carried out by the worker during the visit, just over half of the users reported (51.9%; 95%CI 51.6-52.1) that the workers carried out all five actions ( Figure 2 ), and this was the composite quality indicator for the CHW home visit. 

 In the unadjusted analysis, the probability of receiving a quality home visit from the CHW was significantly higher in the Northeast when compared to the other regions, in municipalities with smaller population sizes (in all groups up to 100,000 inhabitants), with lower and higher FHS coverage, among teams that have defined their catchment area, that considered risk and vulnerability criteria when defining the number of people under their responsibility, among teams that monitor and analyze health indicators and information, that evaluate user satisfaction and that do not have an undiscovered population of workers. The likelihood of the worker’s visit being of adequate quality was even higher among the younger population, those of mixed race, brown, black, and other skin colors, and among those users who have someone at home with walking difficulties ( Table 3 ). 

**Table 3 -:** Prevalence, Prevalence Ratio (PR), and Confidence Interval (95%CI) of adequate quality of the visit carried out by the health worker, according to the characteristics of the municipalities, teams, and individuals (n = 112,741). PMAQ* Cycle 3. Brazil, 2017-2018

**Variable**	**Prevalence**	**Crude PR (95%CI)** ^†^		**p-value**	**Adjusted PR (IC95%)** ^†^		**p-value**
**Level 1**							
Region ^‡^				**<0,001**	-	**-**	**-**
North East	57.2	1.19	1.18-1.21		-	-	
North	48.5	1.01	0.99-1.04		-	-	
Midwest	49.6	1.03	1.01-1.06		-	-	
Southeast	47.9	1.00	-		-	-	
South	49.3	1.03	1.01-105		-	-	
**Level 2**							
Size of municipality (inhabitants) ^§^				**<0.001**			**<0.001**
Up to 10,000	52.1	1.06	1.03-1.08		1.06	1.03-1.09	
10,001-30,000	53.2	1.08	1.06-1.10		1.05	1.03-1.07	
30,001-100,000	53.6	1.09	1.07-1.11		1.07	1.05-1.10	
**Level 2**							
100,001-300,000	49.3	1.00	-		1.00	-	
More than 300,000	49.4	1.00	0.98-1.02		0.97	0.94-0.99	
Family health coverage (%) ^§^				**<0.001**			**<0.001**
Up to 50	54.5	1.15	1.13-1.18		1.20	1.18-1.23	
50.1-75.0	47.2	1.00	-		1.00	-	
75.1-99.9	49.1	1.04	1.02-1.06		0.97	0.95-0.99	
100.0	53.9	1.14	1.12-1.16		1.03	0.99-1.04	
**Level 3**							
Definition of the team’s catchment area ^‡^				**<0.001**			**0.001**
No	43.4	1.00	-		1.00	-	
Yes	51.9	1.20	1.11-1.29		1.15	1.06-1.25	
Consideration of risk and vulnerability criteria to define the number of people under the team’s responsibility ^‡^				**<0.001**			**<0.001**
No	47.7	1.00	-		1.00	-	
Yes	53.3	1.12	1.10-1.13		1.05	1.04-1.07	
Monitoring and analysis of health indicators and information ^‡^				**<0.001**			**<0.001**
No	44.2	1.00	-		1.00	-	
Yes	52.8	1.19	1.17-1.22		1.14	1.12-1.17	
User satisfaction assessment ^‡^				**<0.001**			**<0.001**
No	45.3	1.00	-		1.00	-	
Yes	52.7	1.16	1.14-1.17		1.11	1.08-1.13	
Population uncovered by health workers ^‡^				**<0.001**			**<0.001**
No	52.7	1.04	1.03-1.06		1.03	1.01-1.04	
Yes	50.5	1.00	-		1.00	-	
**Level 4**							
Sex ^†^				0.148			0.507
Male	51.5	1.00	-		1.00	-	
Female	52.0	1.01	0.99-1.02		1.00	0.99-1.01	
Age (years) ^§^				**0.001**			0.479
60 or more	50.2	1.00	-		1.00	-	
40-59	52.9	1.06	1.04-1.07		1.04	1.02-1.05	
18-39	51.9	1.03	1.02-1.05		1.00	0.99-1.02	
Skin color/ethnicity ^‡^				**<0.001**			0.927
White	51.0	1.00	-		1.00	-	
Indigenous/Brown/Black/Other	52.3	1.02	1.01-1.04		1.00	0.99-1.02	
**Level 5**							
Chronic Condition ^‡^				0.443			**<0.001**
Hypertension and diabetes	52.7	1.02	1.00-1.04		1.02	1.01-1.05	
Hypertension or diabetes	51.7	1.00	-		1.00	-	
None	51.9	1.00	0.99-1.02		0.99	0.97-1.00	
**Level 5**							
Someone with walking difficulties in the house ^‡^				**0.001**			**<0.001**
Yes	53.6	1.04	1.02-1.06		1.05	1.02-1.07	
No	51.7	1.00	-		1.00	-	
Pregnancy in the last 2 years ^‡^				0.226			0.393
Yes	51.6	1.00	-		1.00	-	
No	52.1	1.01	0.99-1.03		0.99	0.97-1.01	
Child up to 2 years old ^‡^				0.193			0.710
Yes	51.3	1.00	-		1.00	-	
No	51.9	1.01	0.99-1.03		1.01	0.97-1.04	

^*^ PMAQ Program for Improving Access and Quality

**^†^ Crude PR and Adjusted PR :**Gross and Adjusted Prevalence Ratio - Poisson Regression

^‡^ Heterogeneity test

^§^ Wald test

 In the adjusted analysis according to the hierarchical model, the Northeast region was 19% more likely to have adequate quality in CHW visits compared to the Southeast region. The probability of adequate quality increased as the population size of the municipalities decreased, being around 6% higher in municipalities with fewer than 100,000 inhabitants when compared to municipalities with 100,000 to 300,000 inhabitants and 20.0% higher in those with lower FHS coverage. Having more FHS coverage lost its association after adjustments ( Table 3 ). 

 Teams that defined their catchment area, monitored and analyzed health indicators and information, and assessed user satisfaction were 11.0 to 15.0% more likely to have a composite quality indicator for CHW visits. Teams that considered risk and vulnerability criteria when defining the number of people under their responsibility and which did not have an undiscovered population of workers were 5% and 3% more likely to have adequate quality ( Table 3 ). 

 After adjustments, there was no significant difference in the composite indicator of the quality of CHW visits by sex, age group, and skin color/ethnicity. The probability of adequate quality of the worker’s visit was 2% higher among users with hypertension and diabetes and 5% higher among users who had someone at home with walking difficulties ( Table 3 ). 

## Discussion

The quality of the CHW visit, as verified by the composite indicator, reached 51.9% in Brazil and was significantly higher in the Northeast, in smaller municipalities, among teams with a defined area, which evaluated indicators and user satisfaction, which used risk and vulnerability criteria to define the number of people under their responsibility, without areas uncovered by workers, among users with chronic illnesses and with some residents at home with walking difficulties.

 In order to guarantee the quality of the CHW’s visits, the high proportion (40%) of teams with a population without CHWs is worrying ^(^ 21 ^)^ . According to data from the latest National Health Survey, there was an increase in the percentage of households that never received visits in the 12 months prior to data collection, from 18% in 2013 to 24% in 2019. The households that reported a monthly visit last year fell from 47% in 2013 to 38% in 2019 ^(^ 5 ^)^ . The insufficient number of workers in the territory can overload those who work there, which affects the quality of the work process. 

 Several studies have shown the work overload faced by health workers due to the complexity of their activities, including duties outside their scope of action ^(^ 17 ^,^ 22 ^)^ . The worker’s work sometimes includes bureaucratic and support tasks for health centers, such as sorting and organizing users’ medical records, working at the reception desk, organizing forms and queues, making phone calls, feeding information systems and even cleaning and disinfection activities, which the worker himself considers to be a deviation of function ^(^ 17 ^)^ . As they take on more and more tasks, they may find it difficult to discern what they are responsible for and what their duties are, as well as generating work overload ^(^ 17 ^)^ . 

 A study carried out in São Paulo ^(^ 23 ^)^ showed that, although the worker offers the community a whole range of social support, health professionals and HFS users characterized the worker’s visits as protocol work, focused on the individual rather than the community, and focused on illness rather than health, which is far removed from the proposals for transforming health care. Sometimes, the questions that community workers ask users are focused on a biomedical and curative model, leaving aside the particularities of each individual and each family and disregarding the social and environmental context ^(^ 23 ^)^ . 

 The work of CHWs includes periodic bureaucratic visits, such as updating the register and focusing on productivity, as well as clinical visits to people with a condition who need to be followed up or who have difficulty getting around ^(^ 18 ^)^ . However, their work in the territory should be planned based on priority lines of care, including preventive visits, health education, and collective and community approach actions ^(^ 17 ^)^ . The expanded and complex work process of CHWs makes them important professionals in promoting care, facilitating the population’s access to the health care network, and mediating the transformation of health practices ^(^ 18 ^)^ . 

 The results of our study also showed that the worker provides information about the actions of the health unit of reference, delivers documents, and schedules appointments or exams. The authors describe that this work aims to simplify life for users who work and/or don’t have time ^(^ 23 ^)^ . The results corroborate a study in Minas Gerais ^(^ 24 ^)^ , where actions such as advising the family on how to use health services, making referrals and scheduling appointments and exams were mentioned by most workers (99.6% and 78.8% respectively). A study carried out with CHWs in five remote rural municipalities in the western region of Pará, Brazil, showed that guiding users on how the health unit works is an activity valued by nurses ^(^ 18 ^)^ . 

 In relation to the factors associated with the quality of the worker’s home visits, it was noted that it was higher in the Northeast region and in municipalities with smaller populations, which can be explained by the fact that the prevalence of receiving home visits is higher in these places (84% and 89%, respectively) compared to the South region and municipalities with larger populations (78% and 73%, respectively) ^(^ 21 ^)^ . However, it should be noted that an increase in the number of visits does not guarantee quality, and paying attention to other associated factors is necessary. As for FHS coverage, the results show divergences, with quality being higher in municipalities with higher and lower coverage. 

 The quality of the worker’s visit was higher in teams with a defined coverage area, as recommended by the National Primary Care Policy (PNAB, its acronym in Portuguese) ^(^ 14 ^)^ . The proximity and bond between professionals and the population and families in their area of coverage make it possible to identify health problems and social vulnerabilities ^(^ 18 ^)^ that determine the health-disease process, and this process can be facilitated by mapping the territory and by social participation. 

 The quality of the workers’ visits was higher in teams where management considered risk and vulnerability criteria to define the number of people under their responsibility. Ordinance 2,488/2011, which approves the PNAB ^(^ 19 ^)^ , establishes that home visits should be planned together with the multi-professional team and that risk and vulnerability criteria should be considered so that families with the greatest need are visited more often, adopting an average of one visit per family per month. The new PNAB of 2017 ^(^ 14 ^)^ emphasizes the importance of prioritizing the most vulnerable and epidemiologically at-risk population, however, it does not refer to the frequency and periodicity of visits. In addition, considering that the minimum number of CHWs per team required is one (1) ^(^ 14 ^)^ , many teams have fewer than the necessary number of workers to meet all the demands of the population in the territory and so end up focusing only on those most in need; the other families are left uncovered, which can impact on the quality of visits, and restrict educational practices and the promotion of health equity in the territory. 

 The quality of the worker’s home visits was also higher in the teams that reported monitoring and analyzing health indicators and information, essential activities for decision-making, implementing health actions and policies, and measuring the quality of care provided by the FHS team. In addition, these actions have been considered for financial transfers to municipalities according to the performance of the teams, a strategy adopted to strengthen primary health care ^(^ 25 ^)^ . 

 However, the financial incentive through PMAQ ended in 2019 and was replaced by the PREVINE Brazil Program ^(^ 26 ^)^ . In the previous funding model, there was a different composition of the budget, such as the fixed Basic Health Care Package (PAB, its acronym in Portuguese), the variable PAB with resources for FHS, Family Health Support Center (NASF, its acronym in Portuguese), and others, with fund-to-fund transfer to municipalities with teams that joined the PMAQ, to encourage the resolution of problems in primary care. The PMAQ’s external evaluation process involved the participation of different actors, such as civil society, health professionals, managers, and academic teaching and research institutions; in addition to addressing a set of quality indicators that showed processes for organizing services and the practices of professionals in the team and the territory ^(^ 25 ^,^ 27 ^)^ . 

 Meanwhile, the new evaluation model has produced a setback in the primary care financing model by focusing the incentive on the registration of individuals, the achievement of targets for a reduced number of indicators aimed at a biomedical model, with no defined coverage territory or reference population for the health teams ^(^ 27 ^-^ 28 ^)^ . In addition to the damaging effects on financing primary care for municipalities, these changes could affect the quality of health care ^(^ 27 ^-^ 29 ^)^ . PREVINE Brazil could jeopardize the universality and equity of the SUS by linking the transfer of funds to registered users and the performance of a reduced set of indicators ^(^ 28 ^)^ . It is worth noting that among the PREVINE indicators, there is no mention of evaluating the work of CHWs, which interrupts the evaluation process. Evaluating and monitoring indicators and generating health information that covers the comprehensiveness and complexity of the service and care contributes to improving quality and making the principles of the SUS and attributes of primary care a reality. 

 Assessing user satisfaction was also a factor that contributed to the quality of the worker’s home visit. User satisfaction is essential in the evaluation process; it is an important tool for identifying aspects and situations that interfere positively and negatively the care provided to the population ^(^ 30 ^)^ . The user’s perspective is the best instrument for evaluation and social participation, contributing to the construction of a quality, equitable, and universal public health system that can meet the real needs of its population ^(^ 30 ^)^ . 

 The quality of the worker’s home visits was also higher in teams that did not have a population of workers. Scholars in the field ^(^ 31 ^)^ point out that simply implementing the FHS does not guarantee changes in the health care model, as it is necessary to change the way care is provided and the way professionals work. It is not a change in the form or structure of the model that will guarantee person-centered practices but rather changes in the work process, which must include welcoming, bonding, and accountability as professionals ^(^ 31 ^)^ . 

 Users with chronic conditions and users who live with someone who has difficulty getting around were also associated with an increase in the quality of the worker’s home visits. These results align with the method used to plan home visits, in which the main focus is on monitoring priority groups corresponding to the Ministry of Health’s programmatic actions, including hypertensive and diabetic patients and those who are bedridden or housebound ^(^ 32 ^)^ . This result indicates the importance of the worker in promoting equity in health, but it is necessary to reflect on whether or not these results reinforce the presence of disease-centered care. 

 This study found no significant differences in the composite indicator of the quality of CHW visits according to users’ sociodemographic factors, which reinforces that CHW home visits promote equity not only in access to services ^(^ 21 ^)^ but also in the quality of health care provided. The worker is a facilitator of the relationship between the user and access to health services, i.e., they can be the main link for vulnerable populations and thus be the protagonist of care that considers the principles of comprehensiveness ^(^ 18 ^)^ . 

 The CHW has been considered an active and effective participant in constructing a universal and comprehensive SUS, which, through its work, materializes and strengthens the attributes of primary care ^(^ 33 ^)^ , acting not only as a support for carrying out educational actions in health. Their participation as members of primary care teams should not be an option but an obligation, as they are part of the historical struggle to guarantee the right to comprehensive health care in the SUS ^(^ 33 ^)^ . 

 However, changes to the PNAB 2017 regarding the configuration of the FHS team could lead community workers to become a dying professional category due to the reduced number of workers linked to each team and the de-characterization of their attributions and educational work ^(^ 22 ^)^ . These issues can interrupt links with users, follow-up, and the implementation of educational and preventive actions, as well as foster inequalities in health access and quality. Given the current context, there is a need to debate the changes proposed by the current PNAB - regarding the incorporation of CHWs into the teams, with the prospect of major disruptions to health practices in the territories - and to monitor the results of these changes on the health of the population, especially the most vulnerable. 

The limitations of this study include a possible selection bias, considering that the teams’ adherence to the PMAQ is a voluntary decision. The quality of the workers’ home visits may be even more precarious than the findings of this study since teams with insufficient or precarious working conditions probably did not participate in the program. Furthermore, research is needed to assess the effectiveness and impact of the quality of the work process on the population’s health.

The findings of this study reinforce the need to increase the quality of home visits by community health workers, the importance of organizing the teams’ work process and the process of evaluating indicators to promote the quality of the worker’s visit and to promote the relevance of this professional in promoting health equity.

 It is suggested that progress be made in terms of training and qualification of CHWs to enable them to reflect on the health-disease process and its social determinants, teamwork, conceptions of family, community, and territory, users’ conceptions of freedom and autonomy, and health policies and their objectives. The place that the worker occupies in the team is another point that should be problematized, with a view to making their participation more recognized and valued ^(^ 23 ^)^ . In addition, the involvement of the family health team - physician, nurse, nursing technician, and other professionals from the multi-professional team - in organizing and directing the CHWs’ work process is essential. 

 In the context of organizing the work process and the evaluation process to foster quality of service, the fundamental role of nurses stands out. Throughout history, they have been workers of change in primary care, where territorialization and health care actions stand out in their work process, with a focus on health promotion, prevention, protection, and recovery actions, meeting individual and collective needs ^(^ 16 ^)^ , which is in line with the proposal of the new care model. Faced with the limitations and setbacks contained in the new PNAB ^(^ 14 ^)^ , such as the flexibilization of the number of CHWs, it is up to the nurse, together with the multi-professional team, to plan and manage care from the perspective of the territory, in order to offer people in all life cycles quality health care, achieving and promoting the principles of the SUS and the attributes of primary care. 

## Conclusion

The composite indicator of the adequate quality of the CHW visit was 51.9%, and the prevalence was higher in the Northeast, in smaller municipalities, among teams that defined an area, evaluated indicators and user satisfaction, and used risk and vulnerability criteria to define the number of people under their responsibility, without an undiscovered population of workers, among users with chronic diseases and among users who had someone at home with mobility difficulties. These results are essential in the management practices and work process of the FHS nurse, who supervises the work of the CHW, leads the team in primary care, and plays a leading role with the multidisciplinary team in strengthening the quality of primary health care.
