# Supplementary material for: Quality of home visits by community health workers in primary care and associated factors
Source: Rev Lat Am Enfermagem. 2024 Nov 25;32:e4398. doi: 10.1590/1518-8345.7203.4398 (PMC11653742; doi:10.1590/1518-8345.7203.4398)
Supplement: Translation

# Translation: Calidad de la visita domiciliaria del agente comunitario de salud en la atención básica y factores asociados *

**DOI:** 10.1590/1518-8345.7203.4397

**Published:** 2024-11-25

**Authors:** Marciane Kessler, Elaine Thumé, Luiz Augusto Facchini, Luiza Carolina Moro, Elaine Tomasi

**Affiliations:** ^1^ Universidade Regional Integrada do Alto Uruguai e das Missões, Curso de Enfermagem, Erechim, RS, Brasil.; ^2^ Becario (a) del Conselho Nacional de Desenvolvimento Científico e Tecnológico (CNPq), Brasil.; ^3^ Universidade Federal de Pelotas, Pelotas, RS, Brasil.; ^4^ Universidade Federal de Pelotas, Departamento de Medicina Social, Pelotas, RS, Brasil.; ^5^ Associação Hospitalar Lenoir Vargas Ferreira, Chapecó, SC, Brasil.

**Keywords:** Estrategia de Salud Familiar, Agentes Comunitarios de Salud, Visita Domiciliaria, Indicadores de Calidad de la Atención de Salud, Equidad en Salud, Enfermería en Salud Comunitaria

## Abstract

evaluar la calidad de la visita domiciliaria del agente comunitario de salud y factores asociados.

estudio transversal con 38.865 equipos y 140.444 usuarios del Programa de Mejora del Acceso y de la Calidad de la Atención Básica. Se estableció la “calidad de la visita domiciliaria” y su asociación con características de los municipios, equipos e individuos, estimada por la razón de prevalencia e intervalos de confianza del 95%.

139.362 (99,2%) usuarios manifestaron que el equipo de salud contaba con agentes comunitarios. La prevalencia de calidad fue del 51,9%. Después de los ajustes, la prevalencia fue mayor en el Nordeste (Razón de Prevalencia: 1,19 [1,18-1,21]), municipios más pequeños (Razón de Prevalencia: 1,06 [1,03-1,09]), entre equipos con definición de área (Razón de Prevalencia: 1,15 [1,06-1,25]), evaluación de indicadores (Razón de Prevalencia: 1,14 [1,12-1,17]) y satisfacción del usuario (Razón de Prevalencia: 1,11 [1,08-1,13]), que consideraron criterios de riesgo y vulnerabilidad para la definición de la cantidad de personas bajo responsabilidad (Razón de Prevalencia: 1,05 [1,04-1,07]) y sin población no cubierta por agentes (Razón de Prevalencia: 1,03 [1,01-1,04]); entre usuarios con enfermedades crónicas (Razón de Prevalencia: 1,02 [1,01-1,05]) y con alguien en el domicilio con dificultades de movilidad (Razón de Prevalencia: 1,05 [1,02-1,07]).

se evidencia la importancia de la organización del proceso de trabajo de los equipos para la calidad de la visita domiciliaria del agente y su papel en la equidad en salud.

## Destacados

**(1)** La mitad de los usuarios recibe visitas domiciliarias del agente con calidad adecuada

**(2)** La calidad es mayor en el Nordeste, en municipios más pequeños y en equipos con definición de área.

**(3)** La calidad aumentó con la evaluación de indicadores y la satisfacción del usuario.

**(4)** Considerar los riesgos y la vulnerabilidad para definir el territorio aumentó la calidad.

**(5)** La calidad fue mayor entre los usuarios con enfermedades crónicas y discapacidades.

## Introducción

 En las últimas dos décadas ha crecido el interés y los esfuerzos por evaluar los avances de la Estrategia de Salud de la Familia (ESF) en Brasil ^(^ 1 ^-^ 5 ^)^ , con el objetivo de evidenciar su eficacia para alcanzar la universalidad, integralidad, longitudinalidad y equidad en salud ^(^ 3 ^)^ , además de demostrar un impacto positivo en la salud de la población. 

 La cantidad de equipos de salud de la familia aumentó de 2.054 en 1998 ^(^ 6 ^)^ a 43.508 en 2019 ^(^ 7 ^)^ . En ese mismo período, la población cubierta por la ESF pasó del 4,4% (siete millones de brasileños) ^(^ 6 ^)^ al 64,2% (134 millones) ^(^ 7 ^)^ , siendo la cobertura superior en las regiones y áreas más pobres de Brasil. La expansión de la ESF en el país contribuyó para que el Sistema Único de Salud (SUS) alcanzara el nivel de estar entre los mayores sistemas públicos de salud del mundo, de acceso universal y basado en la atención primaria ^(^ 8 ^)^ . 

 Paralelamente al crecimiento de la cobertura de la ESF en el territorio nacional, la literatura ha mostrado el impacto del modelo en la disminución de la mortalidad infantil ^(^ 9 ^-^ 10 ^)^ , las hospitalizaciones por condiciones sensibles a la atención primaria ^(^ 10 ^)^ , en el control de enfermedades cardiovasculares ^(^ 11 ^)^ y en la reducción de la desigualdad social en la mortalidad de los ancianos ^(^ 2 ^)^ . La ESF también ha contribuido al aumento de la cobertura de acciones programáticas, como, por ejemplo, el prenatal ^(^ 12 ^)^ , la prevención del cáncer de cuello uterino ^(^ 13 ^)^ y al mayor acceso y utilización de los servicios de salud, especialmente entre las personas socialmente vulnerables ^(^ 1 ^-^ 3 ^)^ . Además, diversas investigaciones han evidenciado la importancia de la ESF para aumentar la calidad de los servicios prestados por la atención primaria ^(^ 3 ^-^ 4 ^)^ . Sin embargo, hasta el momento no se han encontrado estudios nacionales dirigidos a evaluar la calidad de los servicios ofrecidos por los Agentes Comunitarios de Salud (ACS) en las visitas domiciliarias. 

 Se destaca que los resultados positivos de la ESF solo fueron posibles debido a su característica de organización y sustitución del modelo tradicional de atención básica. La ESF tiene un modelo de atención dirigido a la atención universal, integral, continua y equitativa ^(^ 1 ^,^ 3 ^)^ , enfocado en la promoción de la salud, la prevención de enfermedades, el diagnóstico y tratamiento temprano; e incluye al agente comunitario como integrante del equipo, que actúa en un área geográfica delimitada, mediante el registro y seguimiento de la población en el territorio. 

 El número de agentes por equipo debe definirse según las características de la población del territorio, considerando criterios de demografía, epidemiología, economía y cuestiones sociales; y en áreas territoriales dispersas, con presencia de riesgo y vulnerabilidad social, se recomienda una cobertura poblacional del 100% ^(^ 14 ^)^ . En 2020, 257.770 agentes comunitarios formaban parte de la ESF, con una cobertura poblacional del 61,1% en el país ^(^ 7 ^)^ . 

 El ACS, que mantiene una relación estrecha y cotidiana con el equipo de enfermería y es gestionado por el enfermero ^(^ 15 ^-^ 16 ^)^ , es también uno de los responsables de los resultados alcanzados por la ESF, cuando desarrolla un trabajo de calidad, siendo el vínculo entre los servicios de salud y la comunidad, y promoviendo la relación entre los usuarios y los profesionales ^(^ 17 ^-^ 18 ^)^ . Una de las principales actividades del agente es la visita domiciliaria ^(^ 18 ^)^ , por medio de la cual realiza el registro de la familia, contribuye con el equipo en la identificación de áreas y situaciones de riesgo, orienta en la promoción y protección de la salud, moviliza a la comunidad para buscar condiciones favorables para la salud y la participación en grupos, notifica a los servicios sobre las enfermedades que requieren vigilancia, deriva a las personas con necesidades de salud a las unidades, acompaña el tratamiento y la rehabilitación de las personas enfermas en el hogar ^(^ 17 ^-^ 19 ^)^ . 

 La caracterización de la calidad en este estudio fue construida desde la perspectiva de la integralidad o la completitud de las actividades, mediante de un enfoque que considera el todo, los aspectos psicológicos, biológicos y socioculturales, junto con la oferta de acciones humanizadas orientadas a la promoción, la protección, la prevención, la recuperación y la rehabilitación de la salud. Uno de los principios más relevantes del SUS es la integralidad, un atributo esencial en la evaluación de la calidad de la atención primaria de salud (APS) y un espacio preferencial para su expresión ^(^ 4 ^)^ . La calidad de la APS es el resultado de la prestación de servicios bien organizados y gestionados, con el apoyo de una financiación suficiente para proveer insumos adecuados, recursos humanos, infraestructura y medicamentos ^(^ 1 ^,^ 4 ^)^ . 

Ante este contexto, el objetivo es evaluar la calidad de la visita domiciliaria del agente comunitario de salud y los factores asociados.

## Método

### Diseño del estudio

Se trata de un estudio transversal basado en servicios de salud, que integró la fase de Evaluación Externa de los equipos de atención básica en todo el territorio nacional. Utilizó datos del tercer ciclo del Programa de Mejora del Acceso y de la Calidad (PMAQ, en su acrónimo en portugués), establecido por el Ministerio de Salud para apoyar la financiación por desempeño en la atención básica en el período de 2011 a 2018.

El PMAQ fue un programa lanzado en 2011 con el propósito de incentivar a los gestores y equipos a mejorar la calidad de los servicios de salud ofrecidos a los ciudadanos del territorio, mediante incentivos financieros federales para los municipios participantes que alcanzaran una mejora en el estándar de calidad. Para ello, se propuso un conjunto de estrategias de cualificación, seguimiento y evaluación del trabajo de los equipos de salud.

 El estudio fue coordinado por más de 50 instituciones brasileñas de Educación Superior y liderado por: *Fundação Oswaldo Cruz* (FIOCRUZ), *Universidade Federal de Minas Gerais* (UFMG), *Universidade Federal do Rio Grande do Norte* (UFRN), *Universidade Federal de Pelotas* (UFPel), *Universidade Federal da Bahia* (UFBA), *Universidade Federal de Mato Grosso do Sul* (UFMS), *Universidade Federal do Pará* (UFPA), *Universidade Federal do Piauí* (UFPI) y *Universidade Federal de Sergipe* (UFS). 

### Criterios de selección

Se incluyeron municipios, equipos de salud y usuarios de equipos de todo el territorio nacional que se adhirieron de forma voluntaria al PMAQ 2017/18. Se excluyeron los usuarios menores de 18 años, aquellos que asistían a la unidad de salud por primera vez o si hacía más de 12 meses que la habían visitado por última vez.

### Población y muestra

En el ciclo 3 de evaluación del PMAQ se incluyeron 5.324 municipios, 28.939 unidades de atención básica, 38.865 equipos y 140.444 usuarios. Participaron en el estudio un profesional médico, enfermero o dentista de cada equipo, sin ser obligatorio que el respondiente desempeñara el rol de coordinador del equipo. El muestreo de los usuarios fue por conveniencia, seleccionando aquellos que asistían al servicio de salud el día de la visita del entrevistador.

### Variables del estudio

 El desenlace “calidad de la visita domiciliaria realizada por el agente comunitario de salud” se construyó considerando la integralidad de las actividades o su realización de manera completa por los ACS, como se mencionó anteriormente ^(^ 4 ^)^ . En este trabajo, se estructuró un indicador compuesto mediante la respuesta positiva de los usuarios a las siguientes preguntas: “Durante la visita, ¿el agente pregunta sobre los problemas de salud de la familia?; ¿Orienta acciones de cuidado y prevención de enfermedades?; ¿Informa sobre las acciones de la unidad básica de salud/puesto de salud cercano a su hogar?; ¿Entrega documentos como, por ejemplo, citas de consultas y exámenes?; y ¿Realiza acciones de combate al mosquito *Aedes aegypti* ?”. Todas las preguntas tuvieron respuesta dicotómica (sí/no). 

Las variables de exposición utilizadas para verificar la asociación con la calidad adecuada de la visita del agente fueron: a) variables de los municipios (fuente secundaria): región geopolítica (Norte, Nordeste, Centro-Oeste, Sudeste y Sur), tamaño poblacional, en habitantes (hasta 10.000; 10.001-30.000; 30.001-100.000; 100.001-300.000; más de 300.000) y cobertura poblacional de la ESF (hasta 50%; 50,1%-75%; 75,1%-99,9%; 100%); b) variables del equipo (entrevista con profesional): definición del área de cobertura del equipo (sí; no); si la gestión consideró criterios de riesgo y vulnerabilidad para definir la cantidad de personas bajo responsabilidad del equipo (sí; no); si el equipo realiza monitoreo y análisis de indicadores e información de salud (sí; no); si el equipo realiza evaluación de la satisfacción del usuario (sí; no); y si existe población no cubierta por agentes en el territorio (sí; no); y c) variables del individuo (entrevista con el usuario del servicio): sexo (masculino; femenino), edad en años completos (18-39; 40-59; 60 o más), etnicidad/color de piel autodeclarado (indígena/pardo/negro/otro y blanco); condiciones crónicas de salud referidas por diagnóstico médico (ninguna; hipertensión o diabetes; hipertensión y diabetes), familiares con dificultad de locomoción investigada por medio de la pregunta: “¿En su casa hay alguien con dificultad de locomoción que necesita atención domiciliaria?” (sí; no), presencia de embarazo en los últimos dos años investigada mediante la pregunta: “¿Estuvo embarazada en los últimos dos años?” (sí; no) y la presencia de hijos de hasta dos años, mediante la pregunta: “¿Tiene hijos de hasta dos años de edad?” (sí; no).

### Instrumento utilizado para la recolección de datos

 Para el presente estudio se utilizaron los datos procedentes de los Módulos I, II y III del instrumento de recolección del PMAQ Ciclo 3 ^(^ 20 ^)^ . El Módulo I se refiere a la observación en la unidad de salud, con preguntas sobre infraestructura; el Módulo II se refiere a la entrevista con un profesional sobre el proceso de trabajo del equipo de atención primaria y la verificación de documentos en la unidad de salud; y el Módulo III se refiere a la entrevista con usuarios en la unidad de salud evaluada. 

### Recolección de datos

El cuestionario se aplicó en las unidades de salud en una fecha acordada con la gestión municipal, siendo el Módulo I datos obtenidos de la observación del entrevistador, el Módulo II respondido por un profesional médico, enfermero o dentista, y el Módulo III por los usuarios presentes en la unidad el día de la evaluación externa.

 La recolección de datos se llevó a cabo entre 2017 y 2018, por alrededor de mil entrevistadores y supervisores capacitados en todas las unidades de la federación, utilizando un instrumento electrónico en *tablets* , con envío automatizado al Ministerio de Salud. El control de calidad de los datos se realizó mediante la supervisión de la recolección y mediante un validador electrónico que verificaba la consistencia entre las respuestas. 

### Análisis de los datos

 Además de los análisis descriptivos, la prevalencia del desenlace se calculó de acuerdo con las características de los municipios, del equipo y del individuo. El análisis de los factores asociados se realizó utilizando regresión de Poisson con ajuste robusto de varianza para estimar las razones de prevalencias (RP) con sus respectivos intervalos de confianza del 95% (IC95%). En el análisis ajustado, se utilizó un modelo jerárquico basado en la determinación social de la salud, destacando las características del contexto como macrodeterminantes de las variables situadas en niveles intermedios y proximales al desenlace ^(^ 1 ^-^ 2 ^)^ . En el primer nivel se incluyó la variable región; en el segundo nivel, las variables relacionadas con el municipio; en el tercer nivel, las variables relacionadas con el equipo; en el cuarto nivel, aquellas relacionadas con las características demográficas y sociales del individuo; y por último, en el quinto nivel, las condiciones de salud individuales. Se empleó una estrategia de selección hacia atrás ( *backward* ) por niveles jerárquicos, eliminando del modelo todas las variables que presentaron un valor p ≥0,20. El criterio adoptado para definir la categoría de referencia (1,00) de cada variable fue el valor más bajo observado, buscando resaltar la probabilidad de aumento de la ocurrencia del desenlace. La significancia estadística se verificó mediante la prueba de Wald y de heterogeneidad, considerando un nivel del 5%. Para el análisis de los datos se utilizó el paquete estadístico *Stata* 14.0 ( *StataCorp* LP, College Station, Estados Unidos). 

### Aspectos éticos

 El proyecto fue sometido y aprobado por el comité de ética en investigación de la *Universidade Federal de Pelotas* , con el dictamen nº 2.453.320 en 2017, bajo el protocolo 80341517.8.1001.5317. Todos los participantes firmaron el consentimiento informado previo a la participación. Los autores declaran no tener conflictos de interés con respecto al tema de estudio. 

## Resultados

 Del total de usuarios entrevistados, 139.362 (99,2%) manifestaron que el equipo de salud contaba con agentes comunitarios, según la Figura 1 . 

**Figura 1 -:** Proceso de selección de la muestra incluida en el análisis. PMAQ* Ciclo 3. Brasil, 2017-2018

*PMAC = Programa de Mejora del Acceso y de la Calidad (PMAQ, su acrónimo en portugués)

 Entre estos, el 37,3% de los usuarios se concentraba en la región Nordeste y el 33,4% en la región Sudeste; el 40,2% residía en municipios con hasta 30.000 habitantes; el 45,1% en municipios con 100% de cobertura; el 99,2% de los usuarios pertenecía a equipos con definición del área de cobertura del equipo, el 80,0% pertenecía a equipos que consideraron criterios de riesgo y vulnerabilidad para la definición de la cantidad de personas bajo su responsabilidad, el 88,4% pertenecía a equipos que realizaban monitoreo y análisis de los indicadores e información de salud, el 87,0% pertenecía a equipos que realizaban una evaluación de la satisfacción de los usuarios y el 39,8% de los usuarios fueron entrevistados en servicios cuyos equipos presentaban áreas en el territorio no cubiertas por ACS ( Tabla 1 ). 

**Tabla 1 -:** Distribución de la muestra de usuarios según características de los municipios y de los equipos (n = 139.362). PMAQ* Ciclo 3. Brasil, 2017-2018

**Variable**	**Total de la muestra**	
	**n**	**%**
**Total**	139.362	100,0
**Características de los municipios**		
Región		
Sur	19.971	14,3
Sudeste	46.609	33,4
Centro-Oeste	11.723	8,4
Nordeste	51.956	37,3
Norte	9.103	6,5
Tamaño del municipio (habitantes)		
Hasta 10.000	18.198	13,1
10.001-30.000	37.795	27,1
30.001-100.000	33.102	23,8
100.001-300.000	18.783	13,5
Más de 300.000	31.484	22,6
Cobertura de ESF ^†^ (%)		
Hasta 50	20.535	14,7
50,1-75,0	26.734	19,2
75,1-99,9	29.256	21,0
100,0	62.837	45,1
**Características del equipo**		
Definición del área de cobertura del equipo		
Sí	138.222	99,2
No	1.140	0,8
Consideración de criterios de riesgo y vulnerabilidad para definir la cantidad de personas bajo responsabilidad del equipo ^‡^		
Sí	101.270	80,0
No	25.293	20,0
Monitoreo y análisis de los indicadores e información de salud		
Sí	123.154	88,4
No	16.208	11,6
Evaluación de la satisfacción del usuario		
Sí	121.221	87,0
No	18.141	13,0
Población no cubierta por agentes		
Sí	55.478	39,8
No	83.884	60,2

^*^ PMAQ Programa de Mejora del Acceso y de la Calidad

^†^ ESF Estrategia Salud de la Familia

^‡^ 12.799 faltantes/sin respuesta

 Entre las características de los usuarios, el 78,4% eran del sexo femenino, el 77,9% tenía entre 18 y 59 años de edad y el 68,1% tenía piel de color pardo, negro o otro. Entre los usuarios, el 28,3% refirió ser hipertenso o diabético y el 9,1% presentaba ambas condiciones; el 7,8% refirió tener a alguien con dificultad de locomoción en la casa donde reside, el 19,2% de las usuarias refirieron embarazo en los últimos 2 años y el 11,2% de los usuarios refirió tener un(a) hijo(a) de hasta 2 años de edad ( Tabla 2 ). 

**Tabla 2 -:** Distribución de la muestra de usuarios según características individuales de los usuarios (n = 139.362). PMAQ* Ciclo 3, Brasil, 2017-2018

**Variable**	**Total de la muestra**	
	**n**	**%**
**Total**	139.362	100,0
**Características individuales**		
Sexo		
Femenino	109.294	78,4
Masculino	30.068	21,6
Edad (años)		
18-39	60.697	43,6
40-59	47.758	34,3
60 o más	30.907	22,2
Etnicidad/Color de piel ^†^		
Blanco	43.937	31,9
Indígena/Pardo/Negro/Otro	93.645	68,1
Condición Crónica ^‡^		
Ninguna	86.779	62,6
Hipertensión o diabetes	39.276	28,3
Hipertensión y diabetes	12.613	9,1
Alguien con dificultad de locomoción en casa ^§^		
Sí	10.878	7,8
No	128.342	92,2
Embarazo en los últimos 2 años ^ǁ^		
Sí	20.953	19,2
No	88.358	80,8
Hijo(a) de hasta 2 años de edad ^⁋^		
Sí	15.621	11,2
No	123.521	88,8

*PMAC Programa de Mejora del Acceso y de la Calidad

^†^ 1.780 faltantes/sin respuesta

^‡^ 694 faltantes/sin respuesta

^§^ 142 faltantes/sin respuesta

^ǁ^ 30.051 faltantes/sin respuesta

^⁋^ 220 faltantes/sin respuesta

 La recepción de visitas del agente entre los equipos que contaban con este profesional fue evaluada por 137.874 usuarios, y 112.741 (81,2%) manifestaron haber recibido visitas ( Figura 1 ). Entre las acciones realizadas por el agente durante la visita, poco más de la mitad de los usuarios refirieron (51,9%; IC95% 51,6-52,1) que los agentes realizaron las cinco acciones. Este fue el indicador compuesto de calidad para la visita domiciliaria del ACS ( Figura 2 ). 

**Figura 2:** - Proporción de las acciones realizadas durante la visita domiciliaria por el agente comunitario y el indicador compuesto de calidad (n = 112.741). PMAQ* Ciclo 3. Brasil, 2017-2018

^*^ PMAQ = Programa de Mejora del Acceso y de la Calidad

 En el análisis no ajustado, la probabilidad de recibir una VD de calidad por parte del ACS fue significativamente mayor en la región Nordeste en comparación con las demás regiones, en los municipios con menores tamaños poblacionales (en todos los grupos hasta 100 mil habitantes), con menor y mayor cobertura de ESF, entre los equipos que tienen una definición del área de cobertura, que consideraron criterios de riesgo y vulnerabilidad para definir la cantidad de personas bajo su responsabilidad, entre los equipos que realizan monitoreo y análisis de indicadores e información de salud, que realizan evaluación de la satisfacción del usuario y que no tienen población no cubierta por agentes. La probabilidad de recibir una visita de calidad adecuada por parte del agente también fue mayor entre la población más joven, de etnicidad/color de piel indígena, pardo, negro y otro, y entre aquellos usuarios que tienen en su domicilio a algún individuo con dificultades de locomoción ( Tabla 3 ). 

**Tabla 3 -:** Prevalencia, Razón de Prevalencia (RP) e Intervalo de Confianza (IC95%) de calidad adecuada de la visita realizada por el agente, según las características de los municipios, equipos e individuos (n = 112.741). PMAQ ^*^ Ciclo 3. Brasil, 2017-2018

**Variable**	**Prevalencia**	**RP bruta (IC95%)** ^†^		**valor-p**	**RP ajustada (IC95%)** ^†^		**valor-p**
**Nivel 1**							
Región ^‡^				**<0,001**	-	**-**	**-**
Nordeste	57,2	1,19	1,18-1,21		-	-	
Norte	48,5	1,01	0,99-1,04		-	-	
Centro-Oeste	49,6	1,03	1,01-1,06		-	-	
Sudeste	47,9	1,00	-		-	-	
Sur	49,3	1,03	1,01-105		-	-	
**Nivel 2**							
Tamaño del municipio (habitantes) ^§^				**<0,001**			**<0,001**
Hasta 10.000	52,1	1,06	1,03-1,08		1,06	1,03-1,09	
10.001-30.000	53,2	1,08	1,06-1,10		1,05	1,03-1,07	
30.001-100.000	53,6	1,09	1,07-1,11		1,07	1,05-1,10	
**Nivel 2**							
100.001-300.000	49,3	1,00	-		1,00	-	
Más de 300.000	49,4	1,00	0,98-1,02		0,97	0,94-0,99	
Cobertura de salud de la familia (%) ^§^				**<0,001**			**<0,001**
Hasta 50	54,5	1,15	1,13-1,18		1,20	1,18-1,23	
50,1-75,0	47,2	1,00	-		1,00	-	
75,1-99,9	49,1	1,04	1,02-1,06		0,97	0,95-0,99	
100,0	53,9	1,14	1,12-1,16		1,03	0,99-1,04	
**Nivel 3**							
Definición del área de cobertura del equipo ^‡^				**<0,001**			**0,001**
No	43,4	1,00	-		1,00	-	
Sí	51,9	1,20	1,11-1,29		1,15	1,06-1,25	
Consideración de criterios de riesgo y vulnerabilidad para definir la cantidad de personas a cargo del equipo ^‡^				**<0,001**			**<0,001**
No	47,7	1,00	-		1,00	-	
Sí	53,3	1,12	1,10-1,13		1,05	1,04-1,07	
Monitoreo y análisis de los indicadores e información de salud ^‡^				**<0,001**			**<0,001**
No	44,2	1,00	-		1,00	-	
Sí	52,8	1,19	1,17-1,22		1,14	1,12-1,17	
Evaluación de satisfacción del usuario ^‡^				**<0,001**			**<0,001**
No	45,3	1,00	-		1,00	-	
Sí	52,7	1,16	1,14-1,17		1,11	1,08-1,13	
Población no cubierta por agentes ^‡^				**<0,001**			**<0,001**
No	52,7	1,04	1,03-1,06		1,03	1,01-1,04	
Sí	50,5	1,00	-		1,00	-	
**Nivel 4**							
Sexo ^†^				0,148			0,507
Masculino	51,5	1,00	-		1,00	-	
Femenino	52,0	1,01	0,99-1,02		1,00	0,99-1,01	
Edad (años) ^§^				**0,001**			0,479
60 o más	50,2	1,00	-		1,00	-	
40-59	52,9	1,06	1,04-1,07		1,04	1,02-1,05	
18-39	51,9	1,03	1,02-1,05		1,00	0,99-1,02	
Color de piel/etnia ^‡^				**<0,001**			0,927
Blanco	51,0	1,00	-		1,00	-	
Indígena/Pardo/Negra/Otro	52,3	1,02	1,01-1,04		1,00	0,99-1,02	
**Nivel 5**							
Condición Crónica ^‡^				0,443			**<0,001**
Hipertensión y diabetes	52,7	1,02	1,00-1,04		1,02	1,01-1,05	
Hipertensión o diabetes	51,7	1,00	-		1,00	-	
Ninguna	51,9	1,00	0,99-1,02		0,99	0,97-1,00	
**Nivel 5**							
Alguien con dificultad de locomoción en casa ^‡^				**0,001**			**<0,001**
Sí	53,6	1,04	1,02-1,06		1,05	1,02-1,07	
No	51,7	1,00	-		1,00	-	
Embarazo en los últimos 2 años ^‡^				0,226			0.393
Sí	51,6	1,00	-		1,00	-	
No	52,1	1,01	0,99-1,03		0,99	0,97-1,01	
Hijo(a) de hasta 2 años de edad ^‡^				0,193			0,710
Sí	51,3	1,00	-		1,00	-	
No	51,9	1,01	0,99-1,03		1,01	0,97-1,04	

^*^ PMAQ Programa de Mejora del Acceso y de la Calidad

**^†^ RP Bruta y RP Ajustada:** Razón de Prevalencia Bruta y Ajustada - Regresión de Poisson

^‡^ Test de heterogeneidad

^§^ Test de Wald

 En el análisis ajustado según el modelo jerárquico, la región Nordeste presentó un 19% más de probabilidad de tener una calidad adecuada en la visita del ACS, en comparación con la región Sudeste. La probabilidad de una calidad adecuada aumentó a medida que disminuyó el tamaño poblacional de los municipios, siendo aproximadamente un 6% mayor en aquellos municipios con menos de 100.000 habitantes en comparación con los municipios de 100.000 a 300.000 habitantes, y un 20% mayor en aquellos con menor cobertura de ESF. Tener una mayor cobertura de ESF perdió asociación tras los ajustes ( Tabla 3 ). 

 Los equipos que tenían definición del área de cobertura, que realizaban monitoreo y análisis de los indicadores e información de salud, y que realizaban evaluación de la satisfacción del usuario presentaron una probabilidad de entre 11% y 15% mayor de tener el indicador compuesto de calidad en la visita del ACS. Los equipos que consideraron criterios de riesgo y vulnerabilidad para definir la cantidad de personas bajo su responsabilidad y que no tenían población no cubierta por agentes presentaron una probabilidad 5% y 3% mayor de tener una calidad adecuada ( Tabla 3 ). 

 Tras los ajustes, no hubo diferencias significativas en el indicador compuesto de calidad de la visita del ACS según sexo, grupo etario o color de piel. La probabilidad de calidad adecuada en la visita del agente fue un 2% mayor entre los usuarios con hipertensión y diabetes, y un 5% mayor entre los usuarios que tenían a alguien en el hogar con dificultades de locomoción ( Tabla 3 ). 

## Discusión

La calidad de la visita del ACS, verificada mediante el indicador compuesto, alcanzó el 51,9% en Brasil, siendo significativamente mayor en el Nordeste, en municipios más pequeños, entre equipos con definición de área, que realizaban evaluación de indicadores y de la satisfacción del usuario, que utilizaban criterios de riesgo y vulnerabilidad para definir el número de personas bajo su responsabilidad, sin áreas no cubiertas por agentes, entre usuarios con enfermedades crónicas y aquellos que tenían algún residente en el hogar con dificultades de locomoción.

 Para garantizar la calidad de la visita del ACS, resulta preocupante la alta proporción (40%) de equipos que tienen población no cubierta por este profesional ^(^ 21 ^)^ . Según los datos de la última Encuesta Nacional de Salud, hubo un aumento en el porcentaje de hogares que nunca recibieron visitas en los 12 meses anteriores a la recolección de datos, de 18% en 2013 a 24% en 2019. La proporción de hogares que informaron visitas mensuales en el último año disminuyó del 47% en 2013 al 38% en 2019 ^(^ 5 ^)^ . La insuficiente cantidad de agentes en el territorio puede sobrecargar a aquellos que ya están trabajando, lo que afecta la calidad del proceso de trabajo. 

 Diversos estudios han evidenciado la sobrecarga de trabajo que enfrenta el agente debido a la complejidad de las actividades desarrolladas, incluyendo tareas que están fuera de su alcance de actuación ^(^ 17 ^,^ 22 ^)^ . El trabajo del agente a menudo incluye tareas burocráticas y de apoyo a las unidades de salud, como la clasificación y organización de expedientes de los usuarios, atención en la recepción, organización de fichas y filas de atención, realización de llamadas telefónicas, alimentación de sistemas de información, e incluso actividades de limpieza y desinfección, consideradas un desvío de función por el propio agente ^(^ 17 ^)^ . Asumir cada vez más tareas puede llevar al agente a tener dificultades para discernir sus verdaderas atribuciones, generando una sobrecarga de trabajo ^(^ 17 ^)^ . 

 Un estudio realizado en São Paulo ^(^ 23 ^)^ mostró que, aunque el agente ofrezca a la comunidad un soporte social integral, los profesionales de la salud y los usuarios de la ESF caracterizaron las visitas del agente como un trabajo protocolario, centrado en el individuo y no en la colectividad, y enfocado en la enfermedad en lugar de la salud, lo que se aleja de las propuestas de transformación de la atención sanitaria. A menudo, las preguntas que el agente comunitario hace a los usuarios están centradas en un modelo biomédico y curativo, dejando de lado las particularidades individuales y familiares, y desconsiderando el contexto social y ambiental ^(^ 23 ^)^ . 

 El trabajo del ACS incluye visitas periódicas burocráticas como la actualización de registros, centradas en la productividad, así como visitas clínicas dirigidas a personas con alguna enfermedad que requieren seguimiento continuo o tienen dificultades de movilidad ^(^ 18 ^)^ . No obstante, su actuación en el territorio debe planificarse en base a líneas de atención prioritarias, incluyendo visitas preventivas y de educación en salud, así como acciones de abordaje colectivo y comunitario ^(^ 17 ^)^ . El proceso de trabajo ampliado y complejo del ACS lo convierte en un profesional relevante para la promoción de cuidados, facilita el acceso de la población a la red de atención sanitaria y actúa como un mediador en la transformación de las prácticas de salud ^(^ 18 ^)^ . 

 Los resultados de nuestro estudio también mostraron que el agente informa sobre las acciones de la unidad de salud de referencia, entrega documentos y programa citas o exámenes. Autores describen que este trabajo tiene como objetivo facilitar la vida de los usuarios que trabajan y/o no disponen de tiempo ^(^ 23 ^)^ . Estos hallazgos coinciden con un estudio realizado en Minas Gerais ^(^ 24 ^)^ , donde acciones como orientar a la familia sobre el uso de los servicios de salud, derivar y programar consultas y exámenes fueron mencionadas por la gran mayoría de los agentes (99,6% y 78,8% respectivamente). Un estudio realizado con ACS en cinco municipios rurales remotos de la región oeste de Pará, Brasil, evidenció que orientar a los usuarios sobre el funcionamiento de la unidad de salud es una actividad valorada por los enfermeros ^(^ 18 ^)^ . 

 En cuanto a los factores asociados a la calidad de las visitas domiciliarias del agente, se observó que fue mayor en la región Nordeste y en los municipios más pequeños, lo que puede explicarse por la mayor prevalencia de visitas domiciliarias en estas áreas (84% y 89%, respectivamente), en comparación con la región Sur y los municipios más grandes (78% y 73%, respectivamente) ^(^ 21 ^)^ . Sin embargo, es importante destacar que el aumento en la cantidad de visitas no garantiza su calidad, por lo que es necesario prestar atención a otros factores asociados. En relación con la cobertura de ESF, los resultados muestran divergencias, siendo la calidad mayor tanto en los municipios con mayor como con menor cobertura. 

 La calidad de la visita del agente fue mayor en los equipos con definición de área de cobertura, conforme a lo estipulado por la Política Nacional de Atención Básica (PNAB) ^(^ 14 ^)^ . La proximidad y el vínculo entre los profesionales y la población y familias del área de cobertura permiten la identificación de problemas de salud y vulnerabilidades sociales ^(^ 18 ^)^ que influyen en el proceso de salud-enfermedad. Este proceso puede facilitarse mediante el mapeo del territorio y la participación social. 

 La calidad de la visita del agente fue mayor en los equipos donde la gestión consideró criterios de riesgo y vulnerabilidad para definir el número de personas bajo su responsabilidad. La Ordenanza nº 2.488/2011, que aprueba la PNAB ^(^ 19 ^)^ , establece que las visitas domiciliarias deben planificarse con el equipo multiprofesional, considerando criterios de riesgo y vulnerabilidad para que las familias con mayor necesidad sean visitadas con más frecuencia, proponiendo una visita mensual por familia. La nueva PNAB de 2017 ^(^ 14 ^)^ enfatiza la importancia de priorizar a la población más vulnerable y en mayor riesgo epidemiológico, aunque no establece la frecuencia de las visitas. Además, considerando que el número mínimo de ACS por equipo es de uno (1) ^(^ 14 ^)^ , muchas unidades tienen un número de agentes por debajo del necesario para cubrir todas las demandas de la población, lo que las obliga a enfocarse solo en las familias más necesitadas, dejando a otras sin cobertura. Esto puede afectar la calidad de las visitas y limitar las prácticas educativas y la promoción de la equidad en salud en el territorio. 

 La calidad de la visita domiciliaria del agente también fue mayor en los equipos que refirieron monitorear y analizar indicadores e información de salud, actividades esenciales para la toma de decisiones, la implementación de acciones y políticas de salud, y la evaluación de la calidad del cuidado brindado por el equipo de ESF. Además, estas acciones han sido consideradas para la transferencia de recursos financieros a los municipios según el desempeño de los equipos, una estrategia adoptada para fortalecer la atención primaria de salud ^(^ 25 ^)^ . 

 Sin embargo, el incentivo financiero por medio del PMAC finalizó en 2019 y reemplazado por el Programa PREVINE Brasil ^(^ 26 ^)^ . En el modelo de financiamiento anterior, había una composición diferente del presupuesto, como el Presupuesto de Atención Primaria (PAB, acrónimo de “Piso de Atenção Básica”, en portugués) fijo, el PAB variable con recursos para equipo de ESF, Centro de Apoyo a la Salud de la Familia (NASF, acrónimo en portugués) y otros, con transferencias fondo a fondo para los municipios con equipos que se unieron al PMAC, para estimular la resolución de problemas en la atención básica. El proceso de evaluación externa del PMAQ involucraba la participación de diferentes actores, como la sociedad civil, profesionales de la salud, gestores e instituciones académicas de enseñanza e investigación; además, abordaba un conjunto de indicadores de calidad que reflejaban procesos de organización de los servicios y prácticas de los profesionales en el equipo y en el territorio ^(^ 25 ^,^ 27 ^)^ . 

 Mientras tanto, el nuevo modelo de evaluación produjo un retroceso en el financiamiento de la atención primaria al centrar el incentivo en el registro de individuos y en el cumplimiento de metas de un número reducido de indicadores orientados hacia un modelo biomédico, sin territorio de cobertura definido ni población de referencia para los equipos de salud ^(^ 27 ^-^ 28 ^)^ . Además de los efectos perjudiciales sobre el financiamiento de la atención básica para los municipios, estos cambios pueden afectar la calidad de la atención a la salud ^(^ 27 ^-^ 29 ^)^ . El PREVINE Brasil puede perjudicar la universalidad y equidad del SUS al vincular los recursos a los usuarios registrados y a un conjunto limitado de indicadores ^(^ 28 ^)^ . Cabe destacar que entre los indicadores del PREVINE no se menciona la evaluación del trabajo de los ACS, interrumpiendo el proceso de evaluación. Evaluar y monitorear indicadores y generar información de salud que abarque la integralidad y la complejidad del servicio y del cuidado contribuye a mejorar la calidad y la aplicación de los principios del SUS y los atributos de la atención primaria. 

 Evaluar la satisfacción del usuario también fue un factor que contribuyó a la calidad de la visita domiciliaria del agente. La satisfacción del usuario es esencial en el proceso de evaluación; constituye una herramienta importante para identificar aspectos y situaciones que influyen positiva o negativamente en la atención a la población ^(^ 30 ^)^ . La opinión del usuario representa el mejor instrumento de evaluación y participación social, contribuyendo a la construcción de un sistema de salud público de calidad, equitativo y universal, que atienda las verdaderas necesidades de su población ^(^ 30 ^)^ . 

 La calidad de la visita domiciliaria del agente también fue mayor en los equipos que no tenían población sin cobertura de agentes. Los expertos en el área ^(^ 31 ^)^ destacan que simplemente implementar la ESF no garantiza cambios en el modelo de atención a la salud; es necesario modificar la forma de prestación de servicios y la actuación de los profesionales. No es el cambio en la estructura del modelo lo que garantizará prácticas centradas en la persona, sino los cambios en el proceso de trabajo, que deben incluir la acogida, el vínculo y la responsabilidad por parte de los profesionales ^(^ 31 ^)^ . 

 Los usuarios con condiciones crónicas y aquellos que conviven con personas con dificultades de locomoción también estuvieron asociados con un aumento en la calidad de la visita domiciliaria del agente. Estos resultados están en línea con el método de planificación de las visitas domiciliarias, cuyo enfoque principal es el seguimiento de los grupos prioritarios, correspondientes a las acciones programáticas del Ministerio de Salud, entre ellos hipertensos, diabéticos y personas encamadas o domiciliadas ^(^ 32 ^)^ . Este resultado destaca la importancia del agente en la promoción de la equidad en salud, pero plantea la necesidad de reflexionar si estos resultados refuerzan o no una atención centrada en la enfermedad. 

 Este estudio no encontró diferencias significativas en el indicador compuesto de calidad de la visita del ACS según factores sociodemográficos de los usuarios, lo que refuerza que las visitas domiciliarias del ACS promueven la equidad no solo en el acceso a los servicios ^(^ 21 ^)^ , sino también en la calidad de la atención sanitaria prestada. El agente se presenta como facilitador de la relación entre el usuario y el acceso a los servicios de salud, lo que significa que puede ser el principal vínculo de las poblaciones vulnerabilizadas, y así, ser protagonista de cuidados que consideran los principios de integralidad ^(^ 18 ^)^ . 

 El ACS ha sido considerado un participante activo y efectivo en la construcción de un SUS universal e integral, que por medio de su trabajo materializa y fortalece los atributos de la atención primaria ^(^ 33 ^)^ , actuando no solo como soporte para la ejecución de acciones educativas en salud. Su participación como integrante de los equipos de atención básica no debería ser una opción, sino una obligación, ya que forma parte de la lucha histórica por garantizar el derecho integral a la salud en el SUS ^(^ 33 ^)^ . 

 Sin embargo, las modificaciones de la PNAB 2017 en cuanto a la configuración de los equipos de ESF podrían llevar al agente comunitario a convertirse en una categoría profesional en extinción, debido a la reducción del número de agentes vinculados a cada equipo y la pérdida de sus funciones y del trabajo educativo que realiza ^(^ 22 ^)^ . Estas cuestiones podrían interrumpir los vínculos con los usuarios, el seguimiento, la ejecución de acciones educativas y preventivas, además de fomentar inequidades en el acceso y en la calidad de la atención sanitaria. Ante este contexto, se destaca la necesidad de debatir las modificaciones propuestas por la actual PNAB, en relación con la incorporación del ACS en los equipos, considerando las posibles rupturas en las prácticas de salud en los territorios, y de monitorear los resultados de estos cambios en la salud de la población, especialmente la más vulnerable. 

Las limitaciones de este estudio incluyen un posible sesgo de selección, considerando que la adhesión de los equipos al PMAC es una decisión voluntaria. La calidad de la visita domiciliaria del agente podría ser aún más precaria de lo que muestran los resultados de este estudio, ya que es probable que los equipos en condiciones de trabajo insuficientes o precarias no hayan participado en el programa. Además, se necesitan investigaciones que evalúen la efectividad y el impacto de la calidad del proceso de trabajo en la salud de la población.

Los hallazgos de esta investigación refuerzan la necesidad de mejorar la calidad de las visitas domiciliarias del agente comunitario de salud, así como la importancia de la organización del proceso de trabajo de los equipos y el proceso de evaluación de indicadores para fomentar la calidad de la visita del agente y resaltar la relevancia de este profesional en la promoción de la equidad en salud.

 Se sugiere avanzar en la perspectiva de la formación y capacitación de los ACS, para permitir reflexiones sobre el proceso salud-enfermedad y sus determinantes sociales, el trabajo en equipo, las concepciones de familia, comunidad y territorio, y las concepciones de libertad y autonomía de los usuarios, así como las políticas de salud y sus objetivos. El lugar que ocupa el agente en el equipo es otro aspecto que debe ser problematizado, con el objetivo de ofrecer una participación más reconocida y valorada ^(^ 23 ^)^ . Además, es fundamental el involucramiento del equipo de salud de la familia – médicos, enfermeros, técnicos de enfermería y otros profesionales del equipo multiprofesional – en la organización y direccionamiento del proceso de trabajo de los ACS. 

 En el contexto de la organización del proceso de trabajo y evaluación orientada a fomentar la calidad del servicio, se destaca el papel fundamental del enfermero, quien a lo largo de la historia se ha constituido como un agente de cambio en el cuidado de la atención básica, donde las acciones de territorialización y atención sanitaria destacan en su proceso de trabajo, con un enfoque en las acciones de promoción, prevención, protección y recuperación de la salud, atendiendo tanto a las necesidades individuales como colectivas ^(^ 16 ^)^ . Esto coincide con la propuesta del nuevo modelo asistencial. Ante las limitaciones y retrocesos presentes en la nueva PNAB ^(^ 14 ^)^ , como la flexibilización del número de ACS, corresponde al enfermero, junto con el equipo multiprofesional, el desafío de planificar y gestionar la atención desde la perspectiva del territorio, para ofrecer a las personas de todas las etapas de la vida una atención sanitaria de calidad, y así promover los principios del SUS y los atributos de la atención primaria. 

## Conclusión

El indicador compuesto de calidad adecuada de la visita del ACS fue del 51,9%, y la prevalencia fue mayor en el Nordeste, en municipios más pequeños, entre equipos con una definición de área de cobertura, que realizaban evaluación de indicadores y de la satisfacción del usuario, que utilizaban criterios de riesgo y vulnerabilidad para la definición del número de personas bajo su responsabilidad, sin áreas no cubierta por agentes, entre usuarios con enfermedades crónicas y aquellos que tenían a alguien en el hogar con dificultades de locomoción. Estos resultados son esenciales para las prácticas de gestión y el proceso de trabajo del enfermero de la ESF, ya que este es el supervisor del trabajo del ACS, líder del equipo en la atención básica y protagonista, junto con el equipo multidisciplinario, en el fortalecimiento y la calidad de la atención primaria en salud.

# Translation: Qualidade da visita domiciliar do agente comunitário de saúde na atenção básica e fatores associados *

**DOI:** 10.1590/1518-8345.7203.4399

**Published:** 2024-11-25

**Authors:** Marciane Kessler, Elaine Thumé, Luiz Augusto Facchini, Luiza Carolina Moro, Elaine Tomasi

**Affiliations:** ^1^ Universidade Regional Integrada do Alto Uruguai e das Missões, Curso de Enfermagem, Erechim, RS, Brasil.; ^2^ Bolsista do Conselho Nacional de Desenvolvimento Científico e Tecnológico (CNPq), Brasil.; ^3^ Universidade Federal de Pelotas, Pelotas, RS, Brasil.; ^4^ Universidade Federal de Pelotas, Departamento de Medicina Social, Pelotas, RS, Brasil.; ^5^ Associação Hospitalar Lenoir Vargas Ferreira, Chapecó, SC, Brasil.

**Keywords:** Estratégia Saúde da Família, Agentes Comunitários de Saúde, Visita Domiciliar, Indicadores de Qualidade em Assistência à Saúde, Equidade em Saúde, Enfermagem em Saúde Comunitária

## Abstract

avaliar a qualidade da visita domiciliar do agente comunitário de saúde e os fatores associados.

estudo transversal com 38.865 equipes e 140.444 usuários do Programa de Melhoria do Acesso e da Qualidade da Atenção Básica. Estabeleceu-se “qualidade da visita domiciliar” e sua associação com características dos municípios, das equipes e dos indivíduos, estimada pela razão de prevalência e por intervalos de confiança de 95%.

139.362 (99,2%) usuários manifestaram que a equipe de saúde possuía agentes comunitários. A prevalência de qualidade foi de 51,9%. Após ajustes, a prevalência foi maior no Nordeste (Razão Prevalência:1,19 [1,18-1,21]), municípios menores (Razão Prevalência: 1,06 [1,03-1,09]), entre equipes com definição de área (Razão Prevalência: 1,15 [1,06-1,25]), avaliação de indicadores (Razão Prevalência: 1,14 [1,12-1,17]) e satisfação do usuário (Razão Prevalência: 1,11 [1,08-1,13]), que consideraram critérios de risco e de vulnerabilidade para definição da quantidade de pessoas sob responsabilidade (Razão Prevalência: 1,05 [1,04-1,07]) e sem população descoberta de agentes (Razão Prevalência: 1,03 [1,01-1,04]); entre usuários com doenças crônicas (Razão Prevalência: 1,02 [1,01-1,05]) e com alguém no domicílio com dificuldades de locomoção (Razão Prevalência: 1,05 [1,02-1,07]).

evidencia-se a importância da organização do processo de trabalho das equipes para a qualidade da visita domiciliar do agente e seu papel na equidade em saúde.

## Destaques

**(1)** Metade dos usuários recebe visita domiciliar do agente com qualidade adequada.

**(2)** Qualidade é maior no Nordeste, em municípios menores e em equipes com definição de área.

**(3)** Qualidade aumentou com a avaliação de indicadores e a satisfação do usuário.

**(4)** Considerar riscos e vulnerabilidade para definir território aumentou a qualidade.

**(5)** Qualidade foi maior entre usuários com doenças crônicas e incapacidades.

## Introdução

 Nas últimas duas décadas têm crescido o interesse e os esforços para avaliar os avanços da Estratégia Saúde da Família (ESF) no Brasil ^(^ 1 ^-^ 5 ^)^ , objetivando evidenciar a sua eficácia para alcançar a universalidade, integralidade, longitudinalidade e equidade em saúde ^(^ 3 ^)^ , além de demonstrar um positivo impacto na saúde da população. 

 A quantidade de equipes de saúde da família aumentou de 2.054, em 1998 ^(^ 6 ^)^ , para 43.508, em 2019 ^(^ 7 ^)^ . Neste mesmo período, a população coberta pela ESF passou de 4,4% (sete milhões de brasileiros) ^(^ 6 ^)^ para 64,2% (134 milhões) ^(^ 7 ^)^ , sendo a cobertura superior nas regiões e áreas mais pobres do Brasil. A expansão da ESF no país contribuiu para que o Sistema Único de Saúde (SUS) chegasse ao patamar de estar entre os maiores sistemas públicos de saúde do mundo, de acesso universal à saúde e baseado nos cuidados primários ^(^ 8 ^)^ . 

 Concomitantemente com o crescimento da cobertura da ESF no território nacional, a literatura tem mostrado o impacto do modelo na diminuição da mortalidade infantil ^(^ 9 ^-^ 10 ^)^ , das internações por condições sensíveis à atenção primária ^(^ 10 ^)^ , no controle de doenças cardiovasculares ^(^ 11 ^)^ e da desigualdade social na mortalidade em idosos ^(^ 2 ^)^ . A ESF tem contribuído ainda para o aumento da cobertura de ações programáticas, o pré-natal ^(^ 12 ^)^ , a prevenção do câncer do colo do útero ^(^ 13 ^)^ e para o maior acesso e utilização aos serviços de saúde, especialmente entre as pessoas socialmente vulnerabilizadas ^(^ 1 ^-^ 3 ^)^ . Além disso, diversas pesquisas têm evidenciado a importância da ESF para aumentar a qualidade dos serviços prestados pela atenção primária ^(^ 3 ^-^ 4 ^)^ . Porém, até o momento não foram encontrados estudos nacionais direcionados para a avaliação da qualidade dos serviços ofertados pelos Agentes Comunitários de Saúde (ACS) na visita domiciliar. 

 Destaca-se que só foi possível o alcance dos resultados positivos da ESF devido à sua característica de organização e de substituição do modelo tradicional de atenção básica. A ESF tem um modelo de atenção direcionado à atenção universal, integral, contínua e equitativa ^(^ 1 ^,^ 3 ^)^ , com foco na promoção de saúde, na prevenção de doenças, no diagnóstico e no tratamento precoce; e inclui o agente comunitário como integrante da equipe, que atua em uma área geográfica delimitada, com cadastro e acompanhamento da população no território. 

 Além disso, o número de agentes por equipe deverá ser definido conforme as características da população do território, considerando critérios de demografia, epidemiologia, economia e questões sociais; e em áreas territoriais dispersas, com presença de risco e vulnerabilidade social, é recomendada a cobertura populacional de 100% ^(^ 14 ^)^ . Em 2020, 257.770 agentes comunitários faziam parte da ESF, com uma cobertura populacional de 61,1% no país ^(^ 7 ^)^ . 

 O ACS, que mantém forte ligação cotidiana com a equipe de enfermagem e é gerenciado pelo enfermeiro ^(^ 15 ^-^ 16 ^)^ , é também um dos responsáveis pelos resultados alcançados pela ESF - quando desenvolve um trabalho de qualidade, sendo o elo entre os serviços de saúde e a comunidade, que promove vínculo entre usuários e profissionais ^(^ 17 ^-^ 18 ^)^ . Umas das principais atividades do agente é a visita domiciliar ^(^ 18 ^)^ , por meio da qual efetiva o cadastro da família, contribui com a equipe na identificação de áreas e situações de risco, orienta a promoção e a proteção da saúde, mobiliza a comunidade a buscar condições favoráveis à saúde e participação em grupos, notifica aos serviços as doenças que necessitam de vigilância, encaminha pessoas com demanda em saúde às unidades, acompanha o tratamento e reabilitação das pessoas doentes em domicílio ^(^ 17 ^-^ 19 ^)^ . 

 A caracterização da qualidade neste estudo foi construída na perspectiva da integralidade ou da completude das atividades, por meio de uma abordagem que considera o todo, ou seja, os aspectos psicológicos, biológicos e socioculturais, junto à oferta de ações humanizadas voltadas a promoção, a proteção, a prevenção, a recuperação e a reabilitação da saúde. Um dos mais relevantes princípios do SUS é a integralidade, sendo também um atributo essencial na avaliação da qualidade da atenção primária à saúde (APS), que se constitui enquanto um espaço preferencial para sua expressão ^(^ 4 ^)^ . A qualidade da APS é resultado da prestação de serviços bem organizados e gerenciados, com suporte de um financiamento suficiente para prover insumos adequados, recursos humanos, infraestrutura e medicamentos ^(^ 1 ^,^ 4 ^)^ . 

Diante deste contexto, tem-se como objetivo avaliar a qualidade da visita domiciliar do agente comunitário de saúde e os fatores associados.

## Método

### Delineamento do estudo

Trata-se de um estudo transversal com base em serviços de saúde e que integrou a fase de Avaliação Externa das equipes de atenção básica em todo território nacional, utilizando dados do terceiro ciclo do Programa de Melhoria do Acesso e da Qualidade (PMAQ), estabelecido pelo Ministério da Saúde para subsidiar o financiamento por desempenho na atenção básica no período de 2011 a 2018.

O PMAQ foi um programa lançado em 2011 com o intuito de incentivar os gestores e as equipes a melhorar a qualidade dos serviços de saúde oferecidos aos cidadãos do território, mediante incentivo financeiro federal para os municípios participantes que atingissem a melhoria no padrão de qualidade. Para isso, foi proposto um conjunto de estratégias de qualificação, acompanhamento e avaliação do trabalho das equipes de saúde.

Acrescenta-se que o estudo foi coordenado por mais de 50 instituições brasileiras de Ensino Superior e lideradas por: Fundação Oswaldo Cruz (FIOCRUZ), Universidade Federal de Minas Gerais (UFMG), Universidade Federal do Rio Grande do Norte (UFRN), Universidade Federal de Pelotas (UFPel), Universidade Federal da Bahia (UFBA), Universidade Federal do Mato Grosso do Sul (UFMS), Universidade Federal do Pará (UFPA), Universidade Federal do Piauí (UFPI) e Universidade Federal de Sergipe (UFS).

### Critérios de seleção

Foram incluídos municípios, equipes de saúde e usuários de equipes de todo o território nacional que aderiram de forma voluntária ao PMAQ 2017/18. Foram excluídos os usuários com menos de 18 anos de idade, que estavam frequentando a unidade de saúde pela primeira vez ou aqueles que não frequentavam a unidade há mais de 12 meses.

### População e amostra

No Ciclo 3 de avaliação do PMAQ foram incluídos 5.324 municípios, 28.939 unidades de atenção básica, 38.865 equipes e 140.444 usuários. Participaram do estudo um profissional médico ou enfermeiro ou dentista de cada equipe, não sendo obrigatório o exercício de papel de coordenador da equipe pelo respondente. Entre os usuários, a amostragem foi por conveniência, ou seja, aqueles que estivessem frequentando o serviço de saúde no dia da visita do entrevistador.

### Variáveis do estudo

 O desfecho “qualidade da visita domiciliar prestada pelo agente comunitário de saúde” foi construído considerando a integralidade das atividades ou sua realização de modo completo pelos ACS, conforme mencionado anteriormente ^(^ 4 ^)^ . Neste trabalho, estruturou-se um indicador composto por meio da resposta positiva dos usuários às seguintes perguntas: “Durante a visita, o agente pergunta sobre os problemas de saúde da família?; Orienta ações de cuidado e prevenção de doenças?; Informa as ações da unidade básica de saúde/posto de saúde próximo da sua casa?; Entrega documentos como, por exemplo, marcação de consultas e exames?; Realiza ações de combate ao mosquito *Aedes aegypti* ?”. Todas as perguntas tiveram resposta dicotômica (sim/não). 

Já as variáveis de exposição utilizadas para verificar associação com a qualidade adequada da visita do agente foram: a) variáveis dos municípios (fonte secundária) - região geopolítica (Norte, Nordeste, Centro-Oeste, Sudeste e Sul), porte populacional em habitantes (até 10.000; 10.001-30.000; 30.001-100.000; 100.001-300.000; mais de 300.000) e cobertura populacional da ESF (até 50%; 50,1%-75%; 75,1%-99,9%; 100%); b) variáveis da equipe (entrevista com profissional) – definição da área de abrangência da equipe (sim; não), se a gestão considerou critérios de risco e vulnerabilidade para a definição da quantidade de pessoas sob responsabilidade da equipe (sim; não), se a equipe realiza monitoramento e análise dos indicadores e das informações de saúde (sim; não), se a equipe realiza avaliação de satisfação do usuário (sim; não) e se existe população descoberta de ACS no território (sim; não); c) variáveis do indivíduo (entrevista com o usuário do serviço) - sexo (masculino; feminino), idade em anos completos (18-39; 40-59; 60 ou mais), etnia/cor da pele autorreferida (indígena/parda/preta/outra e branca), condições crônicas de saúde referida por diagnóstico médico (nenhuma, hipertensão ou diabetes, hipertensão e diabetes), familiar com dificuldade de locomoção investigada por meio da pergunta: “Na sua casa tem alguém com dificuldade de locomoção e que necessita de atendimento no domicílio?” (sim; não), presença de gravidez nos últimos dois anos investigada pela pergunta: “A senhora ficou grávida nos últimos dois anos?” (sim; não) e a presença de filhos de até dois anos de idade, por meio da pergunta: “O(A) senhor(a) tem filho(a) de até dois anos de idade?” (sim; não).

### Instrumento utilizado para a coleta das informações

 Para o presente estudo foram utilizadas as informações oriundas dos Módulos I, II e III do instrumento de coleta do PMAQ Ciclo 3 ^(^ 20 ^)^ . O Módulo I refere-se à observação na unidade de saúde, com questões sobre infraestrutura; o Módulo II refere-se à entrevista com um profissional sobre o processo de trabalho da equipe de atenção primária e verificação de documentos na unidade de saúde; e o Módulo III refere-se à entrevista com usuários na unidade de saúde avaliada. 

### Coleta de dados

O questionário foi aplicado nas unidades de saúde em data acordada com a gestão municipal, sendo o Módulo I dados decorrentes da observação do entrevistador, o Módulo II respondido por um profissional médico, enfermeiro ou dentista e o Módulo III por usuários presentes na unidade no dia da avaliação externa.

 A coleta de dados foi realizada no período de 2017 a 2018, por cerca de mil entrevistadores e supervisores treinados em todas as unidades da federação, utilizando instrumento eletrônico em *tablets* , com envio automatizado ao Ministério da Saúde. O controle de qualidade dos dados foi feito por meio de supervisão da coleta de dados e de um validador eletrônico com checagem da consistência entre as respostas. 

### Análise dos dados

 Além das análises descritivas, a prevalência do desfecho foi calculada de acordo com as características dos municípios, da equipe e do indivíduo. A análise dos fatores associados foi realizada utilizando a regressão de Poisson, com ajuste robusto de variância para estimar as razões de prevalências (RP) e com seus respectivos intervalos de confiança de 95% (IC95%). Na análise ajustada, utilizou-se um modelo hierárquico fundamentado na determinação social da saúde, valorizando as características do contexto como macrodeterminantes das variáveis situadas em níveis intermediário e proximal ao desfecho ^(^ 1 ^-^ 2 ^)^ . No primeiro nível foi incluída a variável região; no segundo nível, as variáveis relacionadas ao município; no terceiro nível, as variáveis relacionadas à equipe; no quarto, aquelas relacionadas às características demográficas e sociais do indivíduo; e, por fim, no quinto nível, as condições de saúde individuais. Aplicou-se seleção para trás ( *backward* ), por níveis hierárquicos, eliminando do modelo todas as variáveis que apresentaram valor p ≥0,20. O critério adotado para a definir a categoria de referência (1,00) de cada variável foi o menor valor observado, buscando destacar a probabilidade de aumento da ocorrência do desfecho. A significância estatística foi verificada pelo teste de Wald e de heterogeneidade, considerando o nível de 5%. Para as análises dos dados, foi utilizado o pacote estatístico *Stata* 14.0 ( *StataCorp LP* , College Station, Estados Unidos). 

### Aspectos éticos

O projeto foi submetido e aprovado pelo comitê de ética em pesquisa da Universidade Federal de Pelotas, com parecer nº 2.453.320, em 2017, sob o protocolo 80341517.8.1001.5317. Todos os participantes assinaram o termo de consentimento livre e esclarecido. Os autores declaram não haver conflitos de interesse em relação ao tema de estudo.

## Resultados

 Do total de usuários entrevistados, 139.362 (99,2%) manifestaram que a equipe de saúde possuía agentes comunitários, conforme Figura 1 . 

**Figura 1 -:** Processo de seleção da amostra incluída na análise. PMAQ* Ciclo 3. Brasil, 2017-2018

*PMAQ = Programa de Melhoria do Acesso e da Qualidade

 Entre estes, 37,3% estavam concentrados na região Nordeste e 33,4% na região Sudeste; 40,2% residiam em municípios com até 30.000 habitantes; 45,1% em municípios com 100% de cobertura; 99,2% dos usuários pertenciam a equipes com definição da área de abrangência; 80,0% pertenciam a equipes que consideraram critérios de risco e vulnerabilidade para a definição da quantidade de pessoas sob responsabilidade; 88,4% pertenciam a equipes que realizavam monitoramento e análise dos indicadores e das informações de saúde; ainda, 87,0% pertenciam a equipes que realizavam avaliação da satisfação dos usuários; e 39,8% dos usuários foram entrevistados em serviços cujas equipes apresentaram áreas no território descobertas por ACS ( Tabela 1 ). 

**Tabela 1 -:** Distribuição da amostra de usuários conforme características dos municípios e das equipes (n = 139.362). PMAQ ^*^ Ciclo 3. Brasil, 2017-2018

**Variável**	**Total da amostra**	
	**n**	**%**
**Total**	139.362	100,0
**Características dos municípios**		
Região		
Sul	19.971	14,3
Sudeste	46.609	33,4
Centro-oeste	11.723	8,4
Nordeste	51.956	37,3
Norte	9.103	6,5
Porte do município (habitantes)		
Até 10.000	18.198	13,1
10.001-30.000	37.795	27,1
30.001-100.000	33.102	23,8
100.001-300.000	18.783	13,5
Mais de 300.000	31.484	22,6
Cobertura de ESF ^†^ (%)		
Até 50	20.535	14,7
50,1-75,0	26.734	19,2
75,1-99,9	29.256	21,0
100,0	62.837	45,1
**Características da equipe**		
Definição da área de abrangência da equipe		
Sim	138.222	99,2
Não	1.140	0,8
Consideração de critérios de risco e vulnerabilidade para definir o número de pessoas sob responsabilidade da equipe ^‡^		
Sim	101.270	80,0
Não	25.293	20,0
Monitoramento e análise dos indicadores e das informações de saúde		
Sim	123.154	88,4
Não	16.208	11,6
Avaliação de satisfação do usuário		
Sim	121.221	87,0
Não	18.141	13,0
População descoberta de ACS ^§^		
Sim	55.478	39,8
Não	83.884	60,2

^*^ PMAQ Programa de Melhoria do Acesso e da Qualidade

^†^ ESF Estratégia Saúde da Família

^‡^ 12.799 faltantes/sem resposta

^§^ ACS Agentes Comunitários de Saúde

 Dentre as características dos usuários, 78,4% eram do sexo feminino, 77,9% possuíam entre 18 e 59 anos de idade e 68,1% possuíam cor da pele mestiça, parda, preta e outra. Entre os usuários, 28,3% referiram ter hipertensão ou diabetes e 9,1% tinham ambas as condições; 7,8% referiram ter alguém com dificuldade de locomoção na casa onde residem; 19,2% das usuárias referiram gravidez nos últimos dois anos e 11,2% dos usuários referiram possuir filho(a) de até dois anos de idade ( Tabela 2 ). 

**Tabela 2 -:** Distribuição da amostra de usuários conforme características individuais dos usuários (n = 139.362). PMAQ ^*^ Ciclo 3. Brasil, 2017-2018

**Variável**	**Total da amostra**	
	**n**	**%**
**Total**	139.362	100,0
**Características individuais**		
Sexo		
Feminino	109.294	78,4
Masculino	30.068	21,6
Idade (anos)		
18-39	60.697	43,6
40-59	47.758	34,3
60 ou mais	30.907	22,2
Etnia/Cor da pele ^†^		
Branca	43.937	31,9
Indígena/Parda/Preta/Outra	93.645	68,1
Condição crônica ^‡^		
Nenhuma	86.779	62,6
Hipertensão ou diabetes	39.276	28,3
Hipertensão e diabetes	12.613	9,1
Alguém com dificuldade de locomoção na casa ^§^		
Sim	10.878	7,8
Não	128.342	92,2
Gravidez nos últimos dois anos ^ǁ^		
Sim	20.953	19,2
Não	88.358	80,8
Filho(a) de até dois anos de idade ^⁋^		
Sim	15.621	11,2
Não	123.521	88,8

*PMAQ Programa de Melhoria do Acesso e da Qualidade

^†^ 1.780 faltantes/sem resposta

^‡^ 694 faltantes/sem resposta

^§^ 142 faltantes/sem resposta

^ǁ^ 30.051 faltantes/sem resposta

^⁋^ 220 faltantes/sem resposta

 O recebimento de visita do ACS, entre equipes que possuíam este profissional, foi avaliado por 137.874 usuários e 112.741 (81,2%) manifestaram receber visitas ( Figura 1 ). Entre as ações realizadas durante a visita, pouco mais de metade dos usuários referiram (51,9%; IC95%51,6-52,1) que os agentes realizaram todas as cinco ações ( Figura 2 ), e este foi o indicador composto de qualidade para a visita domiciliar do ACS. 

**Figura 2 -:** Proporção das ações realizadas na visita domiciliar pelo agente comunitário e indicador composto de qualidade (n = 112.741). PMAQ ^*^ Ciclo 3. Brasil, 2017-2018

^*^ PMAQ = Programa de Melhoria do Acesso e da Qualidade

 Na análise não ajustada, a probabilidade de receber uma visita domiciliar de qualidade do ACS foi significativamente maior na região Nordeste, quando comparada às demais regiões; nos municípios com menores portes populacionais (em todos os grupos até 100 mil hab.) com menor e maior cobertura de ESF; entre as equipes que possuem definição da área de abrangência que consideraram critérios de risco e vulnerabilidade para a definição da quantidade de pessoas sob responsabilidade; entre as equipes que realizam monitoramento e análise dos indicadores e das informações de saúde, que realizam avaliação de satisfação do usuário e que não possuem população descoberta de ACS. A probabilidade de qualidade adequada da visita do ACS ainda foi maior entre a população mais jovem, etnia/cor da pele indígena, parda, preta e outra, e entre aqueles usuários que possuem no algum indivíduo com dificuldades de locomoção no domicílio ( Tabela 3 ). 

**Tabela 3 -:** Prevalência, Razão de Prevalência (RP) e Intervalo de Confiança (IC95%) de qualidade adequada da visita realizada pelo agente, conforme características dos municípios, das equipes e dos indivíduos (n = 112.741). PMAQ ^*^ Ciclo 3. Brasil, 2017-2018

**Variável**	**Prevalência**	**RP bruta (IC95%)** ^†^		**valor-p**	**RP ajustada (IC95%)** ^†^		**valor-p**
**Nível 1**							
Região ^‡^				**<0,001**	-	**-**	**-**
Nordeste	57,2	1,19	1,18-1,21		-	-	
Norte	48,5	1,01	0,99-1,04		-	-	
Centro-oeste	49,6	1,03	1,01-1,06		-	-	
Sudeste	47,9	1,00	-		-	-	
Sul	49,3	1,03	1,01-105		-	-	
**Nível 2**							
Porte do município (habitantes) ^§^				**<0,001**			**<0,001**
Até 10.000	52,1	1,06	1,03-1,08		1,06	1,03-1,09	
10.001-30.000	53,2	1,08	1,06-1,10		1,05	1,03-1,07	
30.001-100.000	53,6	1,09	1,07-1,11		1,07	1,05-1,10	
100.001-300.000	49,3	1,00	-		1,00	-	
Mais de 300.000	49,4	1,00	0,98-1,02		0,97	0,94-0,99	
Cobertura de saúde da família (%) ^§^				**<0,001**			**<0,001**
Até 50	54,5	1,15	1,13-1,18		1,20	1,18-1,23	
50,1-75,0	47,2	1,00	-		1,00	-	
75,1-99,9	49,1	1,04	1,02-1,06		0,97	0,95-0,99	
100,0	53,9	1,14	1,12-1,16		1,03	0,99-1,04	
**Nível 3**							
Definição da área de abrangência da equipe ^‡^				**<0,001**			**0,001**
Não	43,4	1,00	-		1,00	-	
Sim	51,9	1,20	1,11-1,29		1,15	1,06-1,25	
Consideração de critérios de risco e vulnerabilidade definir número de pessoas sob responsabilidade da equipe ^‡^				**<0,001**			**<0,001**
Não	47,7	1,00	-		1,00	-	
Sim	53,3	1,12	1,10-1,13		1,05	1,04-1,07	
Monitoramento e análise dos indicadores e das informações de saúde ^‡^				**<0,001**			**<0,001**
Não	44,2	1,00	-		1,00	-	
Sim	52,8	1,19	1,17-1,22		1,14	1,12-1,17	
Avaliação de satisfação do usuário ^‡^				**<0,001**			**<0,001**
Não	45,3	1,00	-		1,00	-	
Sim	52,7	1,16	1,14-1,17		1,11	1,08-1,13	
População descoberta de ACS ^‡^				**<0,001**			**<0,001**
Não	52,7	1,04	1,03-1,06		1,03	1,01-1,04	
Sim	50,5	1,00	-		1,00	-	
**Nível 4**							
Sexo ^†^				0,148			0,507
Masculino	51,5	1,00	-		1,00	-	
Feminino	52,0	1,01	0,99-1,02		1,00	0,99-1,01	
Idade (anos) ^§^				**0,001**			0,479
60 ou mais	50,2	1,00	-		1,00	-	
40-59	52,9	1,06	1,04-1,07		1,04	1,02-1,05	
18-39	51,9	1,03	1,02-1,05		1,00	0,99-1,02	
Cor da pele/etnia ^‡^				**<0,001**			0,927
Branca	51,0	1,00	-		1,00	-	
Indígena/Parda/Preta/Outra	52,3	1,02	1,01-1,04		1,00	0,99-1,02	
**Nível 5**							
Condição crônica ^‡^				0,443			**<0,001**
Hipertensão e diabetes	52,7	1,02	1,00-1,04		1,02	1,01-1,05	
Hipertensão ou diabetes	51,7	1,00	-		1,00	-	
Nenhuma	51,9	1,00	0,99-1,02		0,99	0,97-1,00	
Alguém com dificuldade de locomoção na casa ^‡^				**0,001**			**<0,001**
Sim	53,6	1,04	1,02-1,06		1,05	1,02-1,07	
Não	51,7	1,00	-		1,00	-	
Gravidez nos últimos 2 anos ^‡^				0,226			0.393
Sim	51,6	1,00	-		1,00	-	
Não	52,1	1,01	0,99-1,03		0,99	0,97-1,01	
Filho(a) de até 2 anos de idade ^‡^				0,193			0,710
Sim	51,3	1,00	-		1,00	-	
Não	51,9	1,01	0,99-1,03		1,01	0,97-1,04	

^*^ PMAQ Programa de Melhoria do Acesso e da Qualidade

**^†^ RP Bruta e RP Ajustada:** Razão de Prevalência Bruta e Ajustada - Regressão de Poisson

^‡^ Teste de heterogeneidade

^§^ Teste de Wald

 Na análise ajustada, segundo o modelo hierárquico, a região Nordeste apresentou 19% maior probabilidade de possuir qualidade adequada na visita do ACS, comparada à região Sudeste. A probabilidade de qualidade adequada aumentou conforme diminuiu o porte populacional dos municípios, sendo em torno de 6% maior naqueles municípios com menor de 100.000 habitantes, isso quando comparado aos municípios de 100 a 300 mil habitantes, e 20,0% maior naqueles com menor cobertura de ESF. Possuir maior cobertura de ESF perdeu associação após ajustes ( Tabela 3 ). 

 Por sua vez, as equipes que possuíam definição da área de abrangência, que realizavam monitoramento e análise dos indicadores e das informações de saúde e que realizavam avaliação de satisfação do usuário apresentaram probabilidade de 11,0 a 15,0% maior de apresentar o indicador composto de qualidade na visita do ACS. Já as equipes que consideraram critérios de risco e de vulnerabilidade para a definição da quantidade de pessoas sob responsabilidade e que não possuíam população descoberta de agentes apresentaram probabilidades 5% e 3% maiores de qualidade adequada ( Tabela 3 ). 

 Após ajustes, não houve diferença significativa no indicador composto de qualidade da visita do ACS por sexo, faixa etária e cor da pele. A probabilidade de qualidade adequada da visita do agente foi 2% maior entre usuários com hipertensão e diabetes e 5% maior entre usuários que possuíam alguém no domicílio com dificuldades de locomoção ( Tabela 3 ). 

## Discussão

A qualidade da visita do ACS, verificada pelo indicador composto, alcançou 51,9% no Brasil, sendo significativamente maior no Nordeste, em municípios menores, entre equipes com definição de área de abrangência, que realizavam avaliação de indicadores e da satisfação do usuário, que utilizavam critérios de risco e de vulnerabilidade para definir o número de pessoas sob responsabilidade, sem áreas descobertas de ACS, entre usuários com condições crônicas e com algum morador no domicílio com dificuldades de locomoção.

 Para garantia da qualidade da visita do ACS, é preocupante a alta proporção (40%) de equipes que possuem população descoberta deste profissional ^(^ 21 ^)^ . Conforme dados da última Pesquisa Nacional de Saúde, houve um aumento da porcentagem de domicílios que não receberam visitas nos 12 meses anteriores à coleta de dados, de 18%, em 2013, para 24%, em 2019. A proporção de domicílios que referiram visita mensal no último ano diminuiu de 47%, em 2013, para 38%, em 2019 ^(^ 5 ^)^ . A quantidade insuficiente de agentes no território pode sobrecarregar aqueles que nele atuam, o que afeta a qualidade do processo de trabalho. 

 Diversos estudos têm evidenciado a sobrecarga de trabalho enfrentada pelo agente devido à complexidade de atividades desenvolvidas, incluindo atribuições que estão fora de seu escopo de atuação ^(^ 17 ^,^ 22 ^)^ . O trabalho do agente inclui, por vezes, tarefas burocráticas e de apoio às unidades de saúde, como por exemplo, separar e organizar prontuários dos usuários, atuar na recepção e acolhimento, organizar fichas e filas de atendimento, realizar chamadas telefônicas, alimentar sistemas de informação e até mesmo atividades de limpeza e desinfecção, consideradas como um desvio de função pelo próprio agente ^(^ 17 ^)^ . Logo, ao abraçar cada vez mais atividades, poderá ter a dificuldade em discernir o que de fato lhe compete e quais suas atribuições, além de gerar sobrecarga de trabalho ^(^ 17 ^)^ . 

 Estudo realizado em São Paulo ^(^ 23 ^)^ mostrou que, embora o agente oferecesse à comunidade todo um suporte social, os profissionais da saúde e os usuários da ESF caracterizaram suas visitas como um trabalho protocolar, voltado ao indivíduo e não à coletividade, e voltado à doença e não à saúde, o que se afasta das propostas de transformações da assistência em saúde. Por vezes, os questionamentos ofertados pelo agente comunitário aos usuários são focados em um modelo biomédico e curativo, deixando de lado as particularidades individuais e de cada família, desconsiderando, assim, o contexto social e ambiental ^(^ 23 ^)^ . 

 Acrescenta-se que o trabalho do ACS inclui visitas periódicas burocráticas, como a atualização de cadastro, e que têm como foco a produtividade, bem como visitas clínicas voltadas para pessoas com algum agravo que necessite de continuidade no acompanhamento ou que tenham dificuldade de deslocamento ^(^ 18 ^)^ . Entretanto, a sua atuação no território deve ser planejada a partir de linhas de cuidado prioritárias, incluindo também visitas preventivas e de educação em saúde, além de ações de abordagem coletiva e comunitária ^(^ 17 ^)^ . O processo de trabalho ampliado e complexo do ACS o torna um profissional relevante na promoção de cuidados, facilita o acesso da população à rede de atenção à saúde e como um mediador na transformação das práticas de saúde ^(^ 18 ^)^ . 

 Os resultados do presente estudo ainda mostraram que o agente informa as ações da unidade de saúde de referência, entrega documentos e agenda consultas ou exames. Autores descrevem que tal trabalho visa facilitar a vida do usuário que trabalha e/ou não possui tempo ^(^ 23 ^)^ . Os resultados corroboram estudo realizado em Minas Gerais ^(^ 24 ^)^ , no qual ações como orientar a família quanto à utilização dos serviços de saúde, encaminhar e agendar consultas e exames foram mencionados pela grande maioria dos ACS (99,6% e 78,8% respectivamente). Outro estudo realizado com ACS em cinco municípios rurais remotos da região oeste do Pará, Brasil, evidenciou que orientar os usuários sobre o funcionamento da unidade de saúde é uma atividade valorizada pelos enfermeiros ^(^ 18 ^)^ . 

 Em relação aos fatores associados à qualidade das visitas domiciliares do ACS, foi possível notar que ela foi maior na região Nordeste e nos municípios de menor porte populacional, o que pode ser explicado pelo fato da prevalência de recebimento de visita domiciliar ser maior nestes locais (84% e 89%, respectivamente) comparado à região Sul e a municípios de maior porte populacional (78% e 73%, respectivamente) ^(^ 21 ^)^ . Entretanto, é preciso destacar que o aumento da quantidade de visitas não garante qualidade, sendo necessário atentar para os demais fatores associados. Quanto à cobertura de ESF, os resultados mostram divergências, sendo a qualidade maior nos municípios de maior e menor cobertura. 

 Além disso, a qualidade da visita do agente foi maior nas equipes com definição da área de abrangência, conforme preconizado pela Política Nacional de Atenção Básica (PNAB) ^(^ 14 ^)^ . A proximidade e o vínculo dos profissionais com a população e as famílias do território de abrangência possibilitam a identificação de problemas de saúde e de vulnerabilidades sociais ^(^ 18 ^)^ que determinam o processo saúde-doença, e este processo pode ser facilitado pelo mapeamento do território e pela participação social. 

 Outrossim, a qualidade da visita do agente também foi maior nas equipes nas quais a gestão considerou os critérios de risco e de vulnerabilidade para definir o número de população sob sua responsabilidade. A Portaria n° 2.488/2011, referente à PNAB ^(^ 19 ^)^ , estabelece que as visitas domiciliares devem ser planejadas junto com a equipe multiprofissional, levando em consideração os critérios de risco e de vulnerabilidade, para que as famílias com maior necessidade sejam visitadas mais vezes, adotando a média de uma visita mensal por família. A nova PNAB de 2017 ^(^ 14 ^)^ enfatiza a importância de priorizar a população de maior vulnerabilidade e de risco epidemiológico, entretanto, não se refere à frequência e à periodicidade das visitas. Além disso, considerando que a quantidade mínima de ACS por equipe exigida é um (1) ^(^ 14 ^)^ , muitas equipes estão com número de agentes abaixo do necessário para atender a todas as demandas da população do território e, assim, acabam focando somente nos mais necessitados; as demais famílias ficam descobertas, o que pode impactar na qualidade das visitas e restringir as práticas educativas, bem como a promoção da equidade em saúde no território. 

 A qualidade da visita domiciliar do agente também foi maior nas equipes que referiram monitorar e analisar indicadores e informações de saúde, atividades essenciais para a tomada de decisão, implementar ações e políticas de saúde e mensurar a qualidade do cuidado prestado pela equipe da ESF. Além disso, tais ações eram consideradas para os repasses financeiros a municípios, de acordo com o desempenho das equipes, como estratégia para o fortalecimento da atenção primária à saúde ^(^ 25 ^)^ . 

 Entretanto, o incentivo financeiro por meio do PMAQ foi encerrado em 2019 e substituído pelo Programa PREVINE Brasil ^(^ 26 ^)^ . No modelo de financiamento anterior havia uma composição diferente do orçamento, como o Piso de Atenção Básica (PAB) fixo, o PAB variável com recursos para equipes da ESF, Núcleo de Apoio à Saúde da Família e outros, com transferência fundo a fundo para os municípios com equipes que aderiram ao PMAQ, para estimular a resolução de problemas na atenção básica. O processo de avaliação externa do PMAQ envolvia a participação de diferentes atores, como a sociedade civil, profissionais da saúde, gestores e instituições acadêmicas de ensino e pesquisa, além de abordar um conjunto de indicadores de qualidade que evidenciavam processos de organização dos serviços e das práticas dos profissionais na equipe e no território ^(^ 25 ^,^ 27 ^)^ . 

 Enquanto isso, o novo modelo de avaliação produziu um retrocesso no modelo de financiamento da atenção primária ao centrar o incentivo no cadastro de indivíduos, no alcance de metas de um número reduzido de indicadores voltados a um modelo biomédico, sem território de abrangência definido e nem população de referência das equipes de saúde ^(^ 27 ^-^ 28 ^)^ . Além dos efeitos danosos no financiamento da atenção básica para os municípios, estas mudanças podem afetar a qualidade da atenção à saúde ^(^ 27 ^-^ 29 ^)^ . O PREVINE Brasil poderá prejudicar a universalidade e a equidade do SUS ao vincular a transferências dos recursos aos usuários cadastrados e ao desempenho de um reduzido conjunto de indicadores ^(^ 28 ^)^ . Vale destacar que entre os indicadores do PREVINE não há menção sobre avaliação do trabalho do ACS, interrompendo o processo de avaliação. Avaliar e monitorar indicadores e gerar informações de saúde que abrangem a integralidade e a complexidade do serviço e do cuidado contribui para a melhoria da qualidade e efetivação dos princípios do SUS, assim como dos atributos da atenção primária. 

 Ademais, avaliar a satisfação do usuário também foi fator que contribuiu para a qualidade da visita domiciliar do agente. A satisfação do usuário é imprescindível no processo de avaliação, pois se constitui em uma ferramenta importante para identificar aspectos e situações que interferem positivamente e negativamente no atendimento à população ^(^ 30 ^)^ . O olhar do usuário representa o melhor instrumento de avaliação e de participação social, contribuindo para a construção de um sistema de saúde público de qualidade, equitativo e universal, que possa atender às reais necessidades da sua população ^(^ 30 ^)^ . 

 A qualidade da visita domiciliar do agente também foi maior nas equipes que não possuíam população descoberta de agentes. Estudiosos da área ^(^ 31 ^)^ destacam que simplesmente implantar a ESF não garante mudanças no modelo assistencial à saúde, sendo necessário mudar a forma de prestação de cuidado e a forma de atuação dos profissionais. Logo, não é a alteração na forma ou na estrutura do modelo que irá garantir práticas centradas na pessoa, mas sim mudanças no processo de trabalho que deve incluir o acolhimento, o vínculo e a responsabilização enquanto profissionais ^(^ 31 ^)^ . 

 Com efeito, usuários com condições crônicas e aqueles que convivem com alguém com dificuldade de locomoção também estiveram associados ao aumento da qualidade da visita domiciliar do agente. Estes resultados vêm de encontro com o método de planejamento das visitas domiciliares, em que o foco principal de atuação é o acompanhamento dos grupos prioritários, correspondentes às ações programáticas do Ministério da Saúde, como a hipertensão, diabetes e pessoas acamadas ou domiciliadas ^(^ 32 ^)^ . Este resultado sinaliza a importância do agente na promoção da equidade em saúde, mas é preciso refletir se estes resultados reforçam ou não a presença de uma atenção centrada na doença. 

 Este estudo não encontrou diferenças significativas no indicador composto de qualidade da visita do ACS conforme fatores sociodemográficos dos usuários, o que reforça que as visitas domiciliares do ACS promovem a equidade não só do acesso aos serviços ^(^ 21 ^)^ , mas também da qualidade da atenção à saúde prestada. Por conseguinte, o agente se coloca como facilitador da relação entre o usuário e o acesso aos serviços de saúde, ou seja, pode ser o principal vínculo das populações vulnerabilizadas e, assim, ser protagonista de cuidados que consideram os princípios da integralidade ^(^ 18 ^)^ . 

 O ACS tem sido considerado participante ativo e efetivo na construção de um SUS universal e integral que, por meio de seu trabalho, materializa e fortalece os atributos da atenção primária ^(^ 33 ^)^ , atuando não somente como suporte para execução de ações educativas em saúde. Sua participação como integrante das equipes de atenção básica não deveria ser uma opção, mas sim uma obrigação, por fazer parte da luta histórica pela garantia do direito integral à saúde no SUS ^(^ 33 ^)^ . 

 Entretanto, alterações da PNAB 2017, referentes à configuração da equipe de ESF, podem levar o agente comunitário para uma categoria profissional em extinção, devido à diminuição do número de agentes vinculados a cada equipe e pela descaracterização de suas atribuições e do seu trabalho educativo ^(^ 22 ^)^ . Estas questões podem interromper os vínculos com os usuários, o acompanhamento, a efetivação de ações educativas e de prevenção, além de fomentar iniquidades no acesso e na qualidade em saúde. Diante do atual contexto, salienta-se a necessidade de debater as alterações propostas pela atual PNAB - no que diz respeito à incorporação do ACS nas equipes, com a perspectiva de importantes rupturas das práticas de saúde nos territórios - e monitorar os resultados destas mudanças na saúde da população, especialmente àquela mais vulnerável. 

As limitações deste estudo incluem um possível viés de seleção, considerando que a adesão das equipes ao PMAQ é uma decisão voluntária. A qualidade da visita domiciliar do agente pode ser ainda mais precária do que mostram os achados deste estudo, uma vez que equipes em condições de trabalho insuficientes ou precárias provavelmente não participaram do programa. Além disso, são necessárias pesquisas que avaliem a efetividade e o impacto da qualidade do processo de trabalho na saúde da população. Ainda, os achados desta pesquisa reforçam a necessidade de aumentar a qualidade das visitas domiciliares do ACS, a importância da organização do processo de trabalho das equipes e do processo de avaliação de indicadores para fomentar a qualidade da visita do agente e também a relevância deste profissional na promoção da equidade em saúde.

 Sugere-se avançar na perspectiva da formação e da qualificação dos ACS, de forma a possibilitar reflexões sobre o processo saúde-doença e seus determinantes sociais, sobre o trabalho em equipe, sobre as concepções de família, de comunidade e território, sobre as concepções de liberdade e autonomia dos usuários e, por fim, sobre as políticas de saúde e seus objetivos. O lugar que o agente ocupa na equipe é outro ponto que deve ser problematizado, visando oportunizar uma participação mais reconhecida e valorizada ^(^ 23 ^)^ . Além disso, é fundamental o envolvimento da equipe de saúde da família – médico, enfermeiro, técnico de enfermagem e demais profissionais da equipe multiprofissional - na organização e direcionamento do processo de trabalho dos ACS. 

 No contexto da organização do processo de trabalho e do processo de avaliação para fomentar a qualidade do serviço, destaca-se o papel fundamental do enfermeiro que se constituiu ao longo da história como um agente de mudanças no cuidado prestado na atenção básica, de modo que as ações de territorialização e de atenção à saúde sobressaem no processo de trabalho, com foco na promoção, prevenção, proteção e recuperação da saúde, atendendo às necessidade individuais e coletivas ^(^ 16 ^)^ , o que vai ao encontro da proposta do novo modelo assistencial. Diante das limitações e retrocessos que constam na nova PNAB ^(^ 14 ^)^ , como a flexibilização do número de ACS, cabe ao enfermeiro, juntamente com a equipe multiprofissional, o desafio de planejar e de gerenciar o cuidado na perspectiva do território, para ofertar às pessoas em todos os ciclos da vida uma atenção à saúde de qualidade, alcançar e promover os princípios do SUS e os atributos da atenção primária. 

## Conclusão

O indicador composto de qualidade adequada da visita do ACS foi de 51,9% e a prevalência foi maior no Nordeste, em municípios menores, entre equipes com definição de área de abrangência que realizavam avaliação de indicadores e da satisfação do usuário, que utilizavam critérios de risco e de vulnerabilidade para a definição do número de pessoas sob responsabilidade, sem população descoberta de ACS, entre usuários com condições crônicas e entre aqueles que tinham alguém com dificuldades de locomoção no domicílio. Estes resultados são essenciais nas práticas de gestão e no processo de trabalho do enfermeiro da ESF, sendo ele o supervisor do trabalho do ACS, líder da equipe na atenção básica e protagonista junto à equipe multidisciplinar, atuando em prol do fortalecimento e da qualidade da atenção primária à saúde.
